# Witch Nails (*Krt90*^*whnl*^): A spontaneous mouse mutation affecting nail growth and development

**DOI:** 10.1371/journal.pone.0277284

**Published:** 2022-11-14

**Authors:** John P. Sundberg, Hannah Galantino-Homer, Heather Fairfield, Patricia F. Ward-Bailey, Belinda S. Harris, Melissa Berry, C. Herbert Pratt, Nicholas E. Gott, Lesley S. Bechtold, Pauline R. Kaplan, Blythe P. Durbin-Johnson, David M. Rocke, Robert H. Rice

**Affiliations:** 1 The Jackson Laboratory, Bar Harbor, ME, United States of America; 2 New Bolton Center, School of Veterinary Medicine, University of Pennsylvania, Kennett Square, PA, United States of America; 3 Maine Medical Center Research Institute, Scarborough, ME, United States of America; 4 Department of Environmental Toxicology, University of California, Davis, CA, United States of America; 5 Department of Applied Biosciences, University of California, Davis, CA, United States of America; Universitat Wien, AUSTRIA

## Abstract

Numerous single gene mutations identified in humans and mice result in nail deformities with many similarities between the species. A spontaneous, autosomal, recessive mutation called witch nails (*whnl*) is described here where the distal nail matrix and nail bed undergo degenerative changes resulting in formation of an abnormal nail plate causing mice to develop long, curved nails. This mutation arose spontaneously in a colony of MRL/MpJ-*Fas*^*lpr*^/J at The Jackson Laboratory. Homozygous mutant mice are recognizable by 8 weeks of age by their long, curved nails. The *whnl* mutation, mapped on Chromosome 15, is due to a 7-bp insertion identified in the 3’ region of exon 9 in the *Krt90* gene (formerly Riken cDNA *4732456N10Rik*), and is predicted to result in a frameshift that changes serine 476 to arginine and subsequently introduces 36 novel amino acids into the protein before a premature stop codon (p. Ser476ArgfsTer36). By immunohistochemistry the normal KRT90 protein is expressed in the nail matrix and nail bed in control mice where lesions are located in mutant mice. Immunoreactivity toward equine KRT124, the ortholog of mouse KRT90, is restricted to the hoof lamellae (equine hoof wall and lamellae are homologous to the mouse nail plate and nail bed) and the mouse nail bed. Equine laminitis lesions are similar to those observed in this mutant mouse suggesting that the latter may be a useful model for hoof and nail diseases. This first spontaneous mouse mutation affecting the novel *Krt90* gene provides new insight into the normal regulation of the molecular pathways of nail development.

## Introduction

The nail unit is a complex anatomic structure in mammals [1]. Abnormalities of the nail unit, usually evident as deformities in the nail plate, the final product of the nail matrix and adjacent structures, are easily observed in humans, primarily due to their large size. Since the mouse nail is small, abnormalities are often overlooked. Some abnormal nail phenotypes, such as long curled nails (onychogryphosis), were overlooked for decades before being reported, as was the case in the spontaneous hairless and rhino mutant mice, both due to mutations in the mouse hairless gene (*Hr*) [2–4]. This oversight continues as many investigators pay little to no attention to the nail unit, or they assume they will not miss lesions at the time of necropsy. Detailed histopathologic analysis of nails should be part of all mutant mouse phenotypic workups that will define common background lesions or artifacts enabling one to better identify real lesions [5,6]. Analysis of the nail unit is complicated at the histological level due to differential textures of the distal phalangeal bone, soft tissues, and nail plate, all of which make sectioning difficult, resulting in poor quality or non-diagnostic slides.

A range of nail abnormalities described in mice range from long curled nails [4,7] and thin nail plates [8,9] to traumatic destruction of the entire nail unit [10]. In the majority of cases, nail unit abnormalities can be observed in young mice through adulthood affecting all nails on both front and rear feet. Spontaneous nail abnormalities, secondary to rotation of the distal phalanx, were observed to be relatively common in some inbred strains that were largely overlooked until the mice became quite old [6].

Here we describe a spontaneous mutation that arose in an MRL/MpJ-*Fas*^*lpr*^/J production colony due to a defect in a novel keratin gene, *Krt90* [11]. Degenerative changes in the matrix and nail bed resulted in the formation of dystrophic nail plates that grew excessively long and curled, hence the name “witch nails” (*whnl*) for this mutation.

These lesions are similar to those observed in laminitis in horses, a common and debilitating hoof disease initiated by multiple types of systemic or orthopedic disease [12]. The equine hoof wall and lamellae are homologous to the mouse nail plate and nail bed, respectively, although hoof lamellae display intricate folding that increases the epithelial surface area of adhesion between the hoof and distal phalanx as part of the evolutionary adaptation of single digit, unguligrade locomotion [13,14]. Laminitis results in damage to, and varying degrees of functional failure, of the hoof lamellae [12]. Features of equine laminitis include the loss of normal hoof lamellar architecture, acanthosis, hyperkeratosis, dystrophic hoof growth patterns resulting in hoof deformation and the formation of a thickened, orthokeratotic nail bed. The distal phalanx often undergoes osteolysis and rotation within the hoof capsule. In severe cases, separation at the dermal epidermal and/or basal-suprabasal cell junctions result in partial or complete sloughing of the hoof [14–17].The equine ortholog of mouse *Krt90*, *KRT124*, is expressed exclusively in the nail bed equivalent in the horse and is the most abundant type II keratin of this structure, suggesting that it is essential for the maintenance of hoof/nail unit form and function [18,19]. Given the practical and ethical concerns of investigating hoof capsule disease in horses and other large animals of agricultural or veterinary interest, the existence of a mutation in mice that replicates many of the nail unit lesions associated with laminitis would facilitate the investigation of equine laminitis and other nail unit diseases.

## Materials and methods

### Source and management of mice

MRL/MpJ-*Fas*^*lpr*^/J mice (Stock No. 000485) were obtained from The Jackson Laboratory (Bar Harbor, ME). Mice with the nail abnormalities were discovered by an animal care technician (Susan Swana) in this colony. Mutant mice were removed and bred to establish a stable and separate colony by mating homozygous mutant mice. Mice in the pedigree that produced the mutant animals were observed to ensure this mutation was not segregating within the main production colony, confirming that all affected lineages had been removed.

Mice were maintained at The Jackson Laboratory in a humidity-, temperature-, and light cycle (12:12) controlled vivarium under specific pathogen-free conditions and were allowed free access to autoclaved food (NIH 31, 6% fat; LabDiet 5K52, Purina Mills, St. Louis, MO) and acidified water (pH 2.8–3.2). All work was done with the approval of The Jackson Laboratory Animal Care and Use Committee in strict accordance with the recommendations in the Guide for the Care and Use of Laboratory Animals of the National Institutes of Health.

### Genetics and identification of the mutated gene

Mutant mice were crossed with each other and with normal mice from the same litter, demonstrating the trait was passed in a simple Mendelian pattern of inheritance. Small sequence length polymorphism (SSLP) mapping was used to map the *whnl* mutation to the distal end of mouse Chromosome 15. The mutation was identified using whole-genome sequencing as previously described [20].

### Histology, immunohistochemistry and immunoblotting

Mice were euthanized by CO_2_ asphyxiation. Complete necropsies were performed on 4 females and 4 males at 24 weeks of age to define the lesions in the nail as well as in any other organs. Initial screening studies utilized tissues fixed in Bouin’s Solution but in the full necropsies tissues were fixed overnight by immersion in Fekete’s acid-alcohol-formalin solution and then transferred to 70% ethanol until trimmed and processed routinely for histologic evaluation [21]. Serial sections of digits with sagittal sections of the nail unit were immunolabeled using antibodies directed against mouse specific keratins 1, 14, 17, and filaggrin [22] (http://tumor.informatics.jax.org/html/antibodies.html). Nails were studied in a total of 32 mutant and 37 wildtype mice at various ages (Table 1).

**Table 1:** Number of mice from which nails were evaluated histologically.

Age	Male Mutant	Female Mutant	Male Control	Female Control
4 weeks	2	4	3	2
8 weeks	6	4	3	4
12 weeks	6	2	5	10
24 weeks	4	4	5	5
Total	18	14	16	21

After the project was completed, a monoclonal antibody directed against horse KRT124 (the ortholog of mouse KRT90) became available and was tested on wildtype mice. The monoclonal antibody is directed against a 14-mer oligopeptide from the C-terminal region of equine KRT124 (RIISKTSTKRSYRS) [18]. The corresponding C-terminal region peptide of murine KRT90 (RI**V**SKTS**S**TK**K**SYRS) has one inserted serine and two amino acid substitutions (valine to isoleucine and lysine to arginine), as indicated by bold font. Immunoblot analysis of total protein extracts of wildtype male (n = 6) and female (n = 6) digits and haired skin was performed using the anti-equine KRT124 monoclonal antibody and a commercial antibody against KRT14 (clone LL002, Abcam Inc., Cambridge, UK) as previously described [18] and detailed in the legend to Fig 7. This antibody detects equine KRT124 by immunoblotting and immunohistochemistry of hoof lamellar tissue (nail bed homolog) but is not immunoreactive to equine haired skin, hoof coronet (nail matrix homolog), corneal limbus, chestnut (callus structure on equine limbs), tongue, oral mucosa, or unhaired (glabrous) skin [19]. Immunoblots were blocked overnight at room temperature with 5% fish gelatin in 0.1% Tween-20 tris-buffered saline, as for previous studies with this antibody in the horse [19]. This blocking protocol is also employed for the KRT14 and β-actin immunoblot using the same blot after stripping (Fig 7 and S1 Raw images). Paraffin embedded sections were labeled using a Ventana XT autostainer (Tuscon, AZ). Diaminobenzidine (DAB; Sigma, St. Louis, MO) was used as the chromogen.

### Scanning electron microscopy

Whole feet were fixed overnight at 4C in 2.5% glutaraldehyde in 0.1M phosphate buffer (PB), pH 7.2. After rinsing the feet several times in PB, they were post-fixed in 1% osmium tetroxide in PB for 48 hours at 4C, dehydrated in a graded series of ethanol and critical point dried. The feet were mounted and sputter-coated with a 4 nm layer of gold. For scanning electron microscopy and elemental analysis, the feet were examined at 20 kV at a working distance of approximately 15 mm on a Hitachi S3000N VP Scanning Electron Microscope (Hitachi Science Systems, Japan). Selected areas of the foot, including the middle digit nail and dorsal skin of the rear foot, the middle digit toe pad of the front foot, as well as two or three hairs, if hairs were present, from the rear foot, were analyzed for elemental composition using an EDAX X-ray microanalysis system (Mahwah, New Jersey). Samples were examined for an average of at least 300 live seconds to ensure a comprehensive reading was obtained [8,23].

### Nail proteomic sample preparation

The rear feet of normal and affected mice were immersed in 2% sodium dodecyl sulfate (SDS)– 0.1 M sodium phosphate buffer (pH 7), incubated in a boiling water bath for 15 minutes, shipped to the University of California, Davis and stored frozen until use. Upon thawing, the samples were heated in a boiling water bath for 10 minutes, and the nails from the middle three digits (D2-4) of each of the right and left hind feet were removed and scraped clean of debris. Nails from the middle three digits only of the right hind paw of the affected mice were analyzed. Each sample was placed in 0.4 ml of 2% sodium dodecanoate (SD) - 0.05 M NH_4_HCO_3_ and agitated with a small magnetic stirring bar for 10 minutes to remove loosely attached debris. The nails were then given a final scraping to ensure removal of tissue debris and boiled again for 5 minutes in SD–NH_4_HCO_3_ buffer. The samples from normal mice (4 mg) were transferred to 0.4 ml of fresh SD—NH_4_HCO_3_ buffer containing 50 mM dithioerythritol (DTE), held in a boiling water bath for 5 minutes and heated overnight in a 70°C oven. Iodoacetamide was added to 100 mM with stirring for 1 hour in the dark. The samples were adjusted to pH 3 by addition of 8 μl of trifluoroacetic acid and extracted three times with 0.6 ml of ethyl acetate, discarding the upper (organic) phase. The pH was then adjusted back to 8.5 by addition of 2.5 μl of concentrated ammonium hydroxide and 25 μl of 1 M NH_4_HCO_3_. To the samples were then added 40 μg of reductively methylated bovine trypsin [24], and digestion proceeded for 3 days at room temperature with daily additions of 40 μg of trypsin. Samples from affected mice (8 mg) were treated the same but in double the volume. The digestion supernatant was submitted for proteomic analysis, and the insoluble pelleted material was rinsed 5 times with water and analyzed for protein content using ninhydrin after sulfuric acid digestion [25]. The insoluble residue accounted for 18 ± 8% of the total mass.

### Mass spectrometry and protein identification

Essentially as previously described [26], digested peptides from both *Krt90*^*+/+*^ and *Krt90*^*whnl/whnl*^ mouse nails were analyzed by LC-MS/MS with a Thermo Scientific Q Exactive Orbitrap Plus mass spectrometer. Sample data were analyzed using X!Tandem to search a Uniprot mouse database appended to a database of common non-human contaminants (cRAP, common Repository of Adventitious Proteins database, http://www.thegpm.org/crap/). Both were appended to an identical but reversed database for calculating false discovery rates (90,758 proteins total), assuming the digestion enzyme was trypsin. Scaffold version 4.4.3 was used to validate MS/MS based peptide and protein identifications. The peptide decoy false discovery rate was 0.1%, and the protein decoy false discovery rate 1.2%. Since certain peptides can overlap among the keratins, data were analyzed in parallel by exclusive spectral counts to ensure the weighted spectral counts were from the identified protein.

### Mouse nail proteomics data analysis

Spectral count data were transformed using a variance stabilizing transformation for negative binomial data, which takes the form *f*_*θ*_*(x) = ln [x + (x*^*2*^ *+ x/θ)*^*1/2*^ *+ (2θ)*^*-1*^*]*. This transformation, when θ is selected to minimize the correlation between the variance and standard deviation of the transformed data, removes mean-variance dependency from the data so that they may be analyzed using methods that assume constant variance across the range of the data. Data were then analyzed using the Bioconductor package for gene expression analysis limma, version 3.24.15 [27], which fits linear models to each protein separately and then applies empirical Bayes shrinkage to the estimated variances in order to increase power. The application of the above transformation to RNA-Seq data is discussed in Rocke *et al*. [28]. The overall analysis approach is similar to that called “limma-trans” in Sonenson and DeLorenzi [29], which employs the variance stabilizing transformation from the DESeq RNA-Seq analysis package [30]. Analyses were conducted using the statistical software environment R, version 3.2.2 [31].

## Results

### Mode of inheritance and identification of the mutated gene

**Fig 1:**
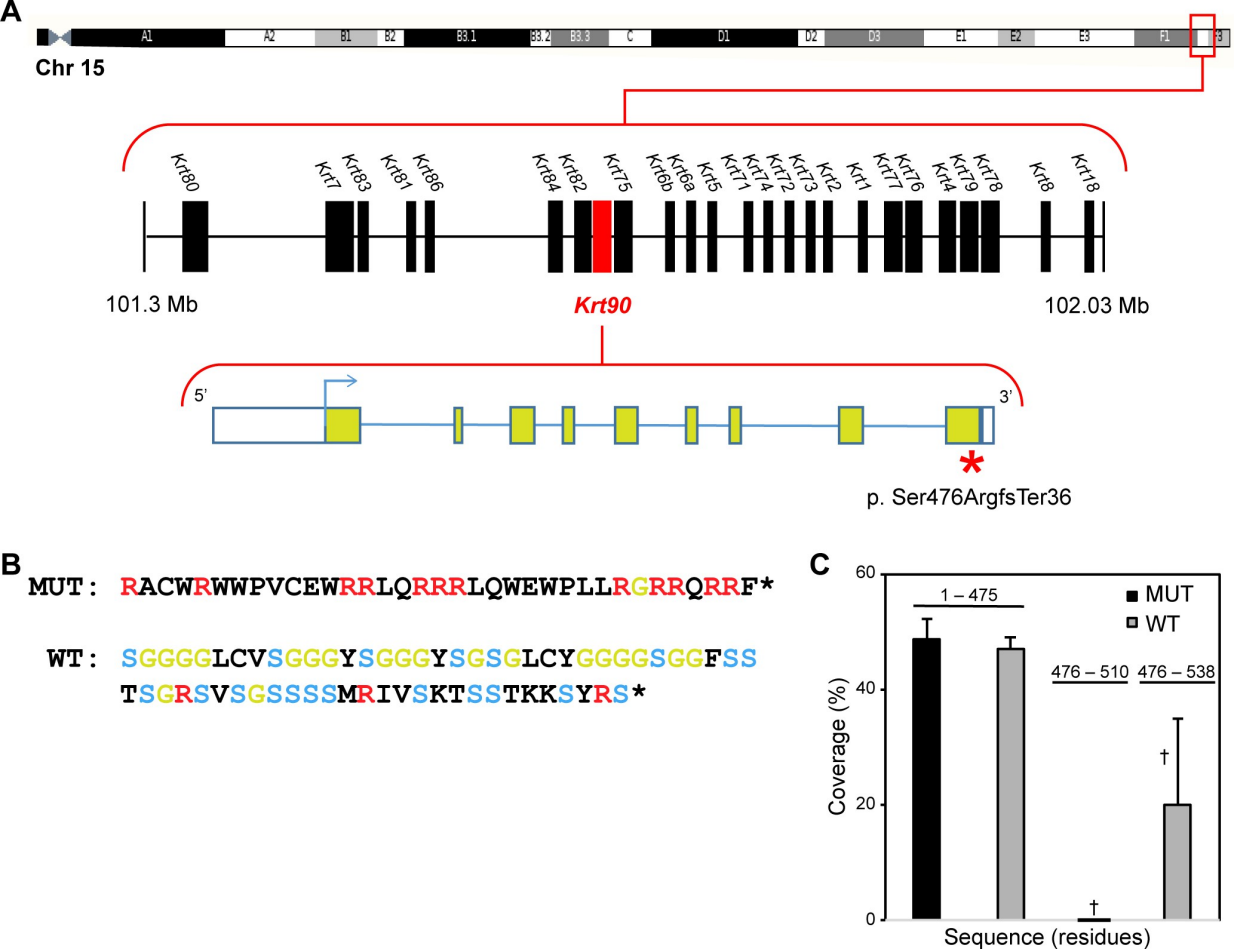
Whole genome sequencing and protein prediction. (A) Whole genome sequencing identified a 7-bp insertion in exon 9 of *Krt90* (formerly the Riken cDNA *4732456N10Rik* gene) resulting in a serine to arginine change at position 476 of the mature protein followed by a frame shift and insertion of 36 novel amino acids and a premature stop codon (left panel). (B) Amino acid sequences of mutant (MUT) and corresponding wild type (WT) C-terminal regions. Residues indicated in color are R (arginine, red), G (glycine, green), and S (serine, blue). (C) Peptide coverage of the mutant and wild type sequences in proteomic analysis, which differ in C-terminal residues (476–510 or 476–538). Yields of the C-terminal regions differed significantly by t-test (†, p = 0.04).

The MRL/MpJ-*Fas*^*lpr*^/J colony carrying the mutated gene was maintained by homozygote x homozygote matings. Affected mice were crossed to CAST/EiJ to map the mutant gene locus and thereby also proving the mode of inheritance. These crosses produced no affected mice in the N1 = F1 generation (no affected/20 born). Of the F2 progeny, 26 affected mice out of 413 were produced (6.3% affected), lower than the expected 25%. No embryos were available for analysis to determine the loss of expected affected progeny. The mutation was mapped using simple sequence length polymorphisms (SSLPs) in F2 progeny to Chromosome 15 between 98.7Mb and 102.8Mb. Whole genome sequencing identified a 7bp insertion in exon 9 of the Riken cDNA *4732456N10Rik* gene [20] (now called Keratin 90, *Krt90*) that resulted in a serine to arginine change at position 476 of the mature protein followed by a frame shift insertion of 36 novel amino acids and a premature stop codon (Fig 1).

### Clinical features

Breeding colonies were set up using MRL/MpJ-*Fas*^*lpr*^/J mice carrying the witch nail phenotype to maintain the colony. The mice are currently cryopreserved and available (B6.MRL-*Krt90*^*whnl*^/GrsrJ, Stock No: 024690, The Jackson Laboratory, Bar Harbor, ME).

**Fig 2:**
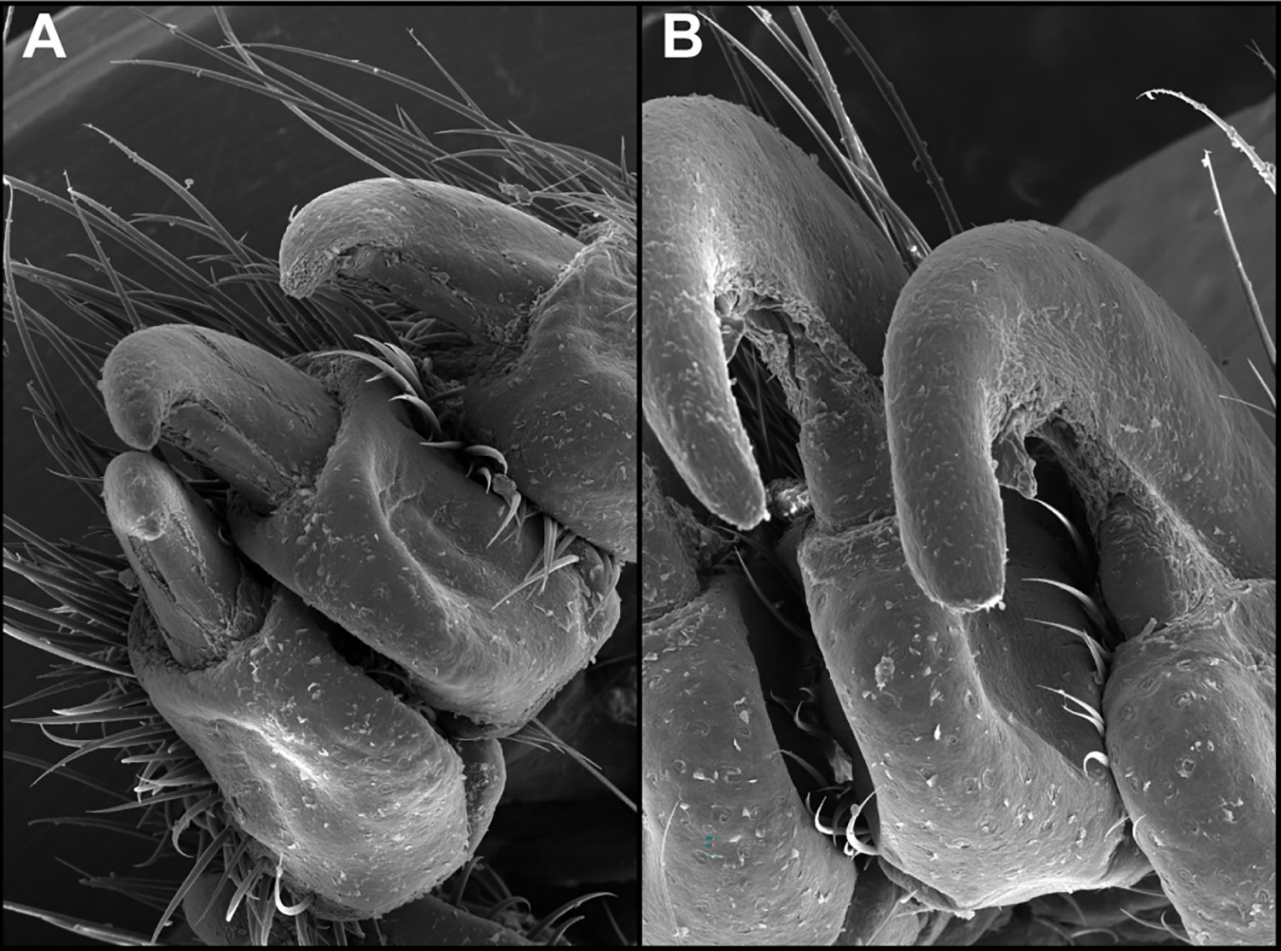
Scanning electron microscopy. Normal wildtype (*Krt90*^*+/+*^; A) nails compared to the long, curved, mutant (*Krt90*^*whnl/whnl*^; B) nails.

The witch nail mutant mice bred normally and had no obvious gross or behavioral abnormalities other than long, curved nails (onychogryphosis) affecting all digits that were first evident at 6 to 8 weeks of age. There was no sexual dichotomy. Details of the long, curved nails were best visualized by scanning electron microscopy (Fig 2). Mutant nails were long and curved due to the abnormal extension of the nail plate, as well as a thickened hyponychium.

### Histology

**Fig 3:**
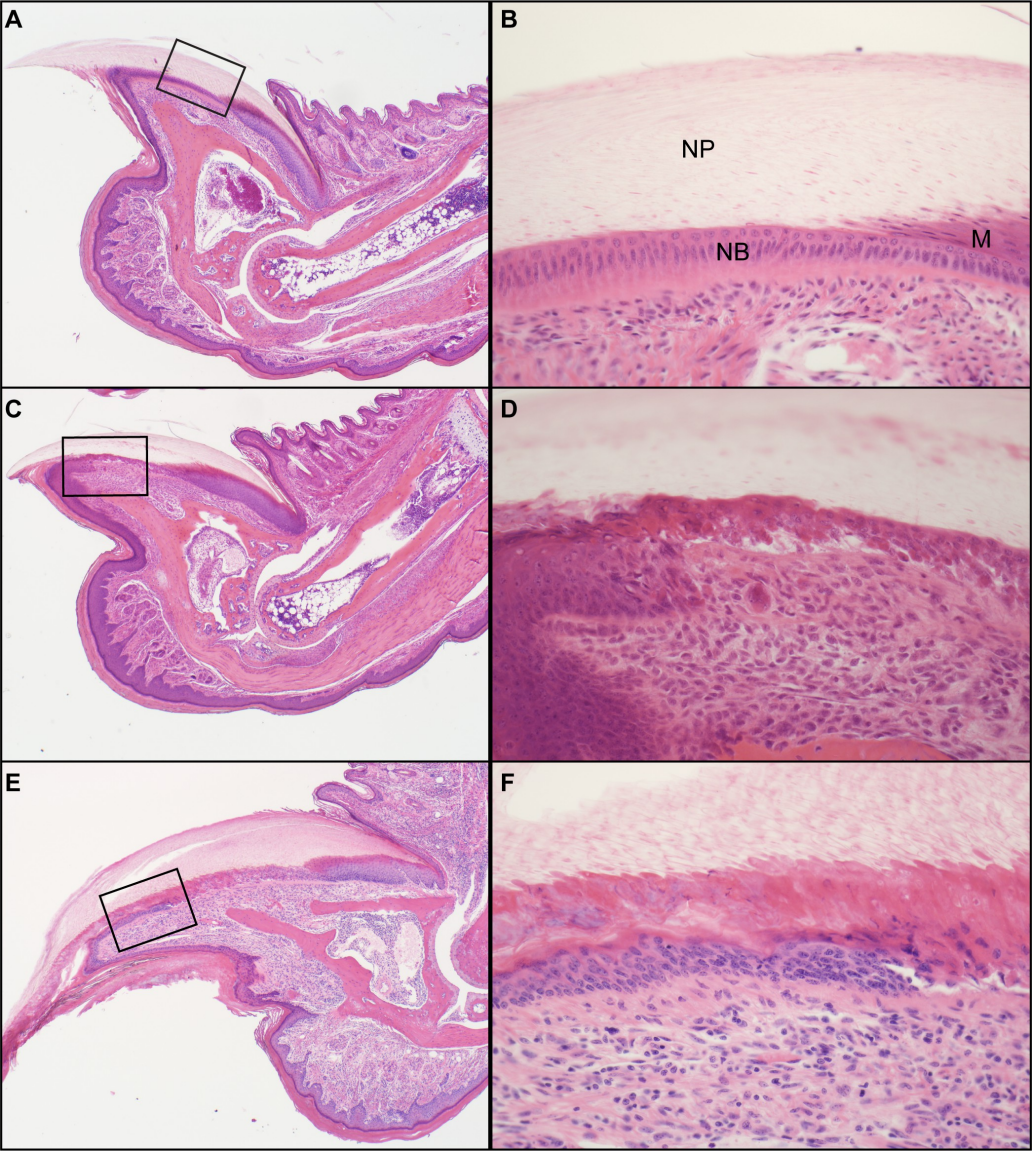
Histologic changes in nails. The wildtype control had a well-formed normal nail plate (NP, A) that covered the nail bed (NB, boxed, B) and matrix (M). By contrast, the nail unit of a 4-week old (C, D) and 24-week old (E, F) *Krt90*^*whnl/whnl*^ mouse had various degrees of nail dystrophy (disorganization of the cells of the nail bed) and premature cornification.

**Fig 4:**
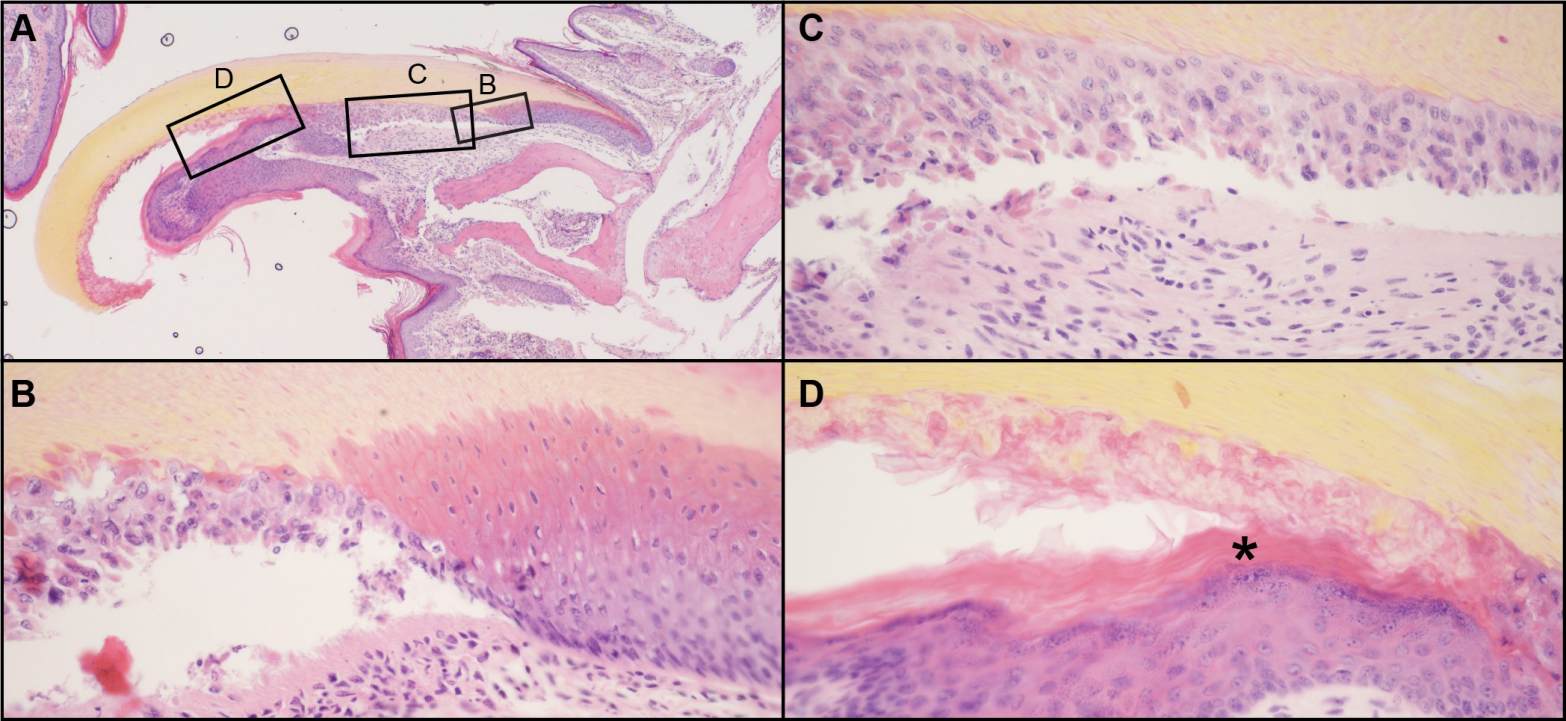
Nail plate separation. A 6-week old male *Krt90*^*whnl/whnl*^ mutant mouse nail section (A) had separation at the dermal-epidermal junction of the nail bed (C) starting at the matrix (B). Premature cornification (*) of the hyponychium extending under the nail bed caused separation of the nail plate from the nail bed (D). Note the abnormal location of the stratum granulosum deep under the nail plate (D, *).

**Fig 5:**
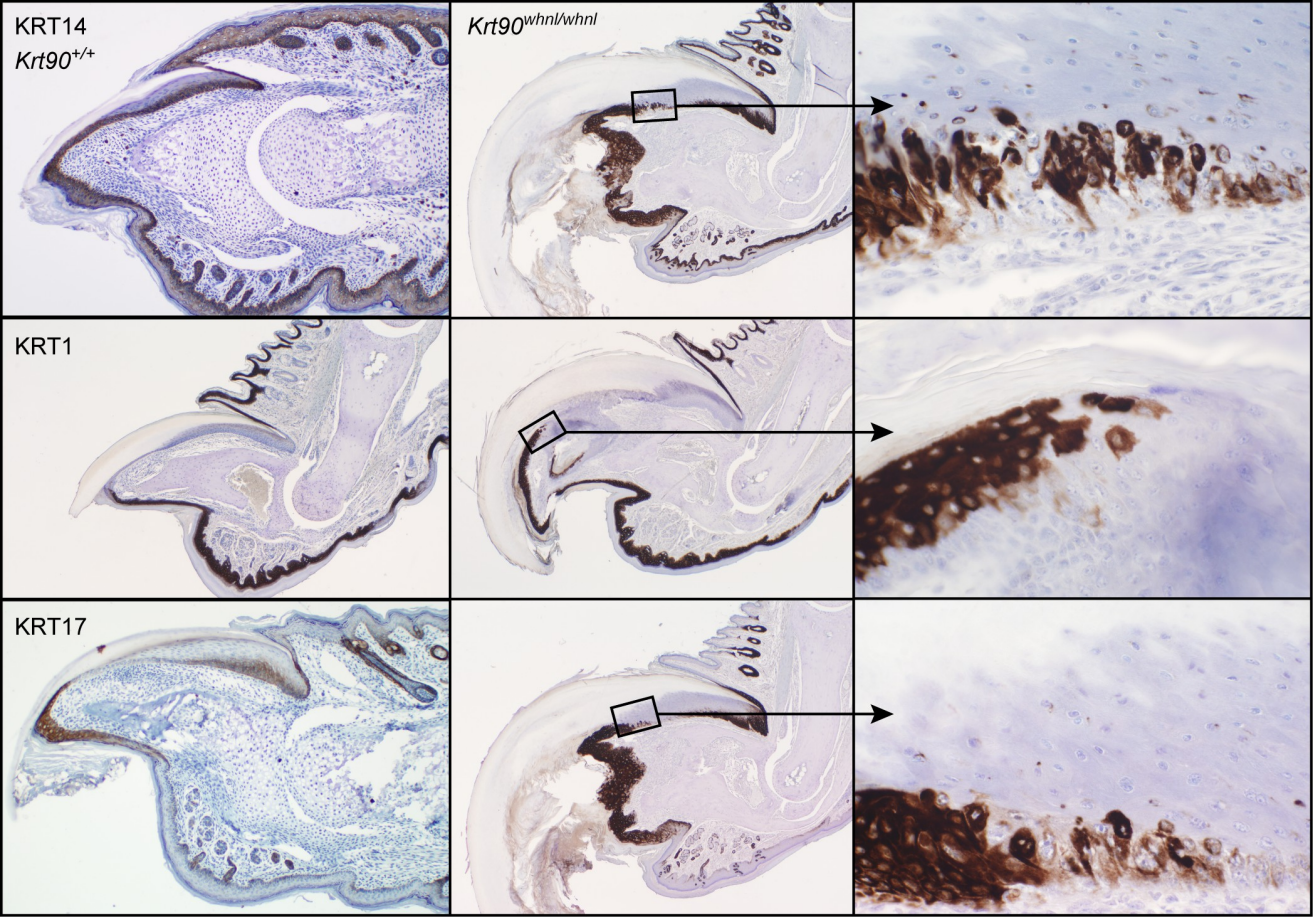
Mouse specific keratins. Mouse specific keratin 14 (KRT14) labeled normal mouse nail matrix, nail bed, and hyponychial basal equivalent cells. Those in the *Krt90*^*whnl/whnl*^ mutant mice had intermittent labeling of cells in the nail bed corresponding to dystrophy and premature cornification. Keratin 1 (KRT1) labeled epidermal suprabasal cells not the nail matrix or nail bed. By contrast, the hyponychium was labeled far back under the nail plate indicating abnormal differentiation. Keratin 17 (KRT17) labeled the hyponychium and part of the matrix. In the *Krt90*^*whnl/whnl*^ mutant mouse, the hyponychium was hyperplastic and extended under the nail bed.

The normal mouse nail unit has a well-organized nail matrix, nail bed, nail plate above the nail bed, and hyponychium (Fig 3A and 3B) [1]. By contrast, the adult nail units of the mutant mice had progressive abnormalities that started at the junction of the nail matrix and nail bed where there was separation at the dermal-epidermal junction (Figs 3C–3F and 4). This separation, which may have been a processing artifact as it was not evident in all cases, continued distally towards the hyponychium. The hyponychium extended further back under the nail plate than normal due to epidermal thickening, cornification, and hypergranulosis (Fig 4D). Hypergranulosis was confirmed by immunohistochemical localization of filaggrin in this area. The cornification resulted in premature separation of the nail plate. Orthokeratotic hyperkeratosis of the hyponychium extended under the nail unit (Figs 3E, 3F and 4D). Mouse specific keratin 14 (KRT14) labeled normal mouse nail matrix, nail bed, and hyponychial basal equivalent cells (Fig 5). Those in the KRT90 mutant mice have intermittent labeling of cells in the nail bed corresponding to necrosis and premature cornification (Fig 5). Keratin 1 (KRT1) labeled epidermal suprabasal cells but not the nail matrix or nail bed (Fig 5). By contrast, in the hyponychium KRT1 labeled far back under the nail plate indicating abnormal differentiation (Fig 5). Keratin 17 (KRT17) labeled the normal hyponychium and part of the matrix. In the KRT90 mutant mouse, the hyponychium was hyperplastic and KRT17 expression extended under the nail bed, similar to KRT1 (Fig 5). Resembling in function the inner root sheath of the hair follicle in separating from the mature hair shaft [32], the observed perturbation might influence the degree of curvature of the nail plate.

**Fig 6:**
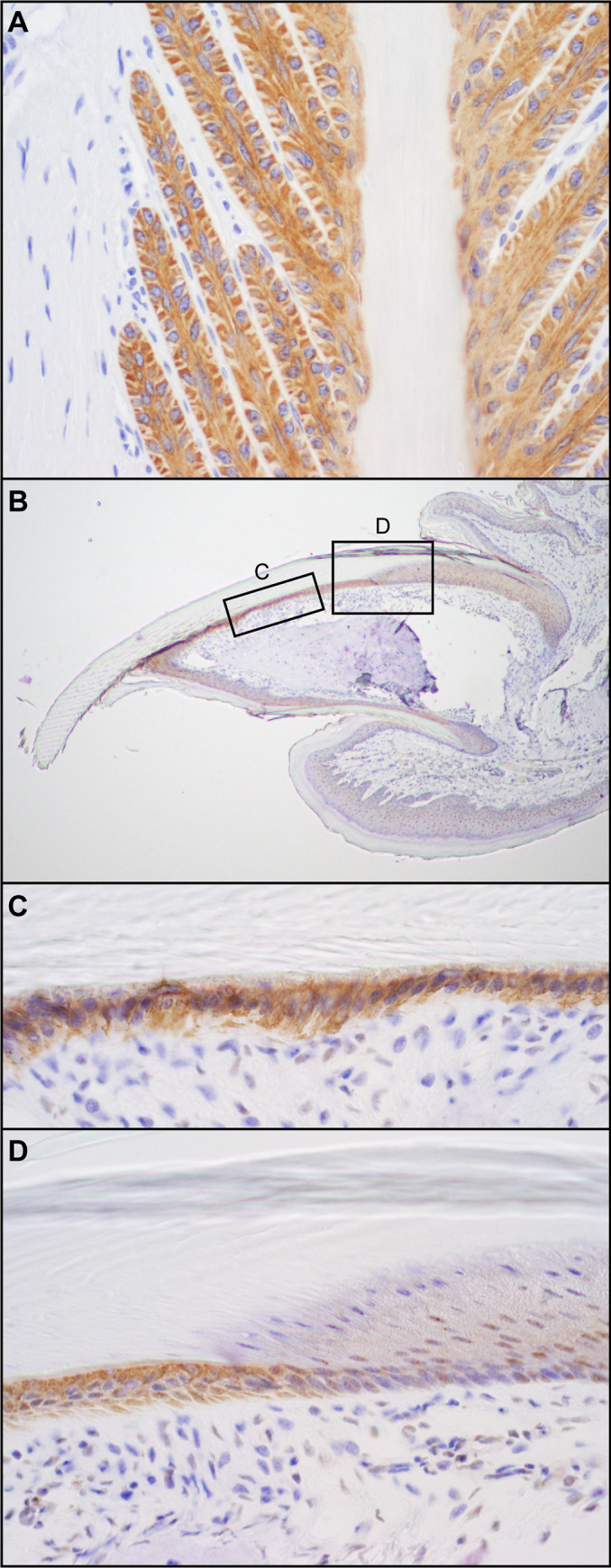
KRT124/KRT90 immunohistochemistry. A monoclonal antibody directed at equine KRT124 (ortholog of mouse KRT90) labeled the secondary epidermal lamellae in a horse hoof (nail bed equivalent, A). The same antibody labeled the nail bed and hyponychium (B-D) of a normal mouse nail unit, the same cells affected in the *Krt90*^*whnl/whnl*^ mutant mouse.

**Fig 7:**
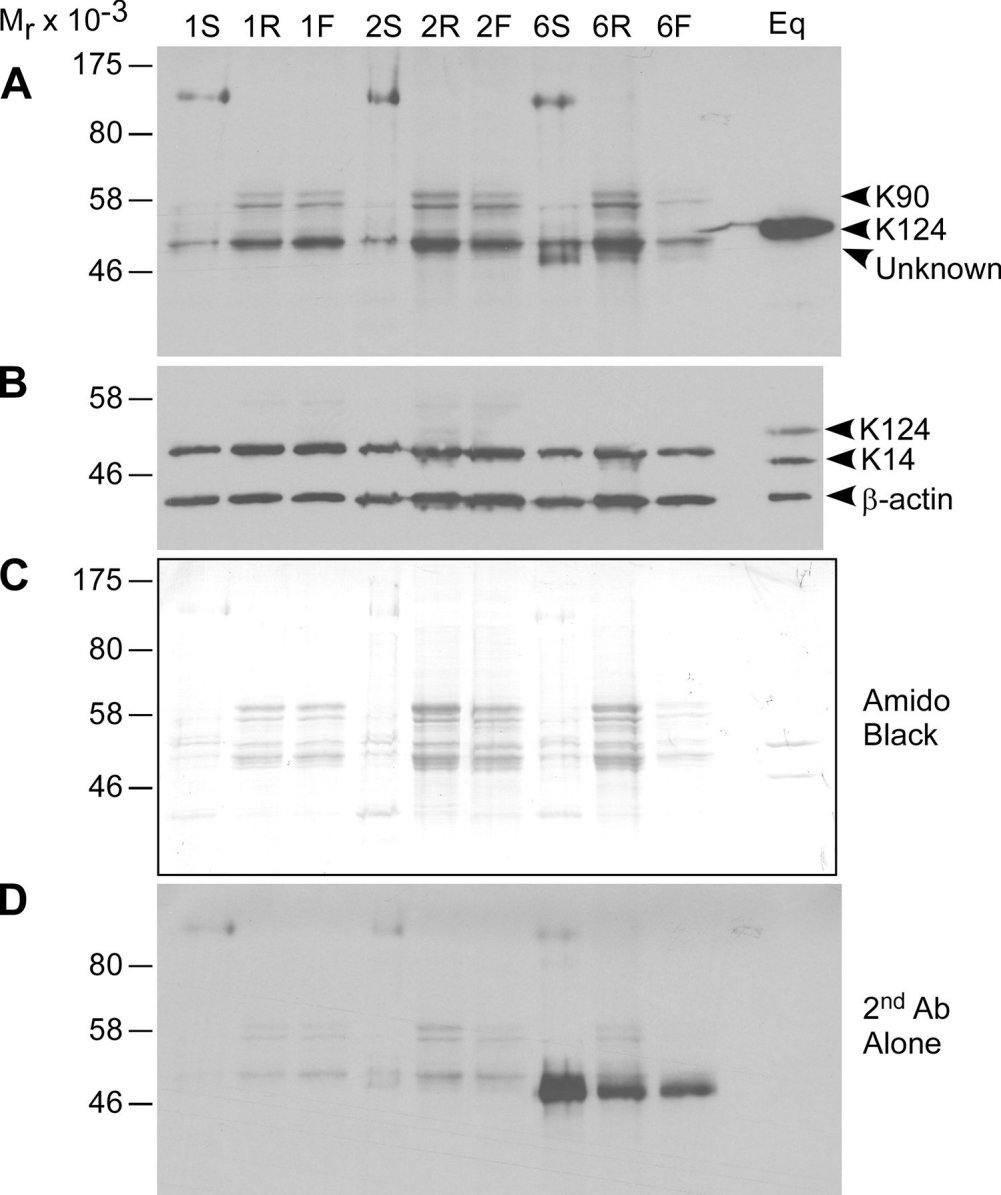
Detection of mouse KRT90 (K90) with anti-equine KRT124 (K124) monoclonal antibody. **A-D:** A single blot from 10% SDS-PAGE (reducing conditions) of protein extracts from haired dorsal skin (lanes 1S, 2S, 6S), left rear toes (lanes 1R, 2R, 6R) and left front toes (1F, 2F, 6F) from wildtype male mice (10 μg total protein) and positive control equine lamellar tissue (Eq; 1 μg total protein) protein extracts. Approximate locations of proteins, based on predicted relative molecular mass (M_r_), indicated to the right. M_r_ of protein standards indicated to the left. Representative images are shown from four experiments using wildtype mouse male (N = 6) and female (N = 6) dorsal skin and toe samples. **A)** Immunoblotting performed using the K124C monoclonal antibody against a C-terminal peptide (1:5,000) as previously described [19]. A single band at approximately 54 kDa was detected in the positive control (Eq) and a doublet was detected at approximately 58 kDa, the predicted M_r_ of murine KRT90 (possibly due to post-translational modification or alternate splicing) in the toe lanes, but not the dorsal skin lanes. There was non-specific detection of an approximately 50 kDa M_r_ band in all mouse protein lanes. This blot was then stripped for 15 min at room temperature using the Restore™ Western Blot Stripping Buffer (Thermo Scientific: Rockford, IL) and re-probed with a mouse anti-human KRT14 monoclonal antibody (1:500; clone LL002, Abcam Inc., Cambridge, UK) and mouse anti-β-actin-HRP mAb (1:15,000, clone AC-15; Sigma-Aldrich: St Louis, MO) as previously described [19]. The equine KRT124 band was still visible at M_r_ 54 kDa due to incomplete stripping and the KRT14 band was at M_r_ 50 kDa and the β-actin band was at approximately M_r_ 42 kDa in both the mouse and equine lanes. The mouse KRT14 bands appeared to align with the non-specific 50 kDa band in **(A)**, consistent with some cross-reactivity of the equine KRT124C antibody with another murine keratin under these conditions**. C)** Following immunoblotting, the blot was stained for protein content using the amido black stain (Sigma-Aldrich). The major protein bands aligned with some of the detected keratin bands, as expected due to the abundance of keratin in skin and nail unit epidermal tissues. **D)** Prior to any immunoblotting, the blot was also probed with the secondary antibody alone and the chemiluminescence exposure was double that shown in **(A)**. Mouse 6 appeared to have immunoreactivity to immunoglobulin in the samples and faint background staining is present in the other lanes, consistent with some cross-reactivity of the secondary antibody with the most abundant proteins.

A monoclonal antibody became available for horse keratin 124, the ortholog of mouse KRT90 [19]. This antibody labeled the secondary epidermal lamellae of a horse hoof (nail bed equivalent; Fig 6A) [14] and detected protein of the predicted relative molecular mass for KRT90 (58 kDa) by immunoblotting of protein extracts from mouse toes, but not dorsal haired skin (Fig 7).The same antibody labeled the nail bed and hyponychium (Fig 6B–6D) of a normal mouse nail unit, the same cells affected in the *Krt90*^*whnl/whnl*^ mutant mouse.

### Proteomic analysis

**Fig 8:**
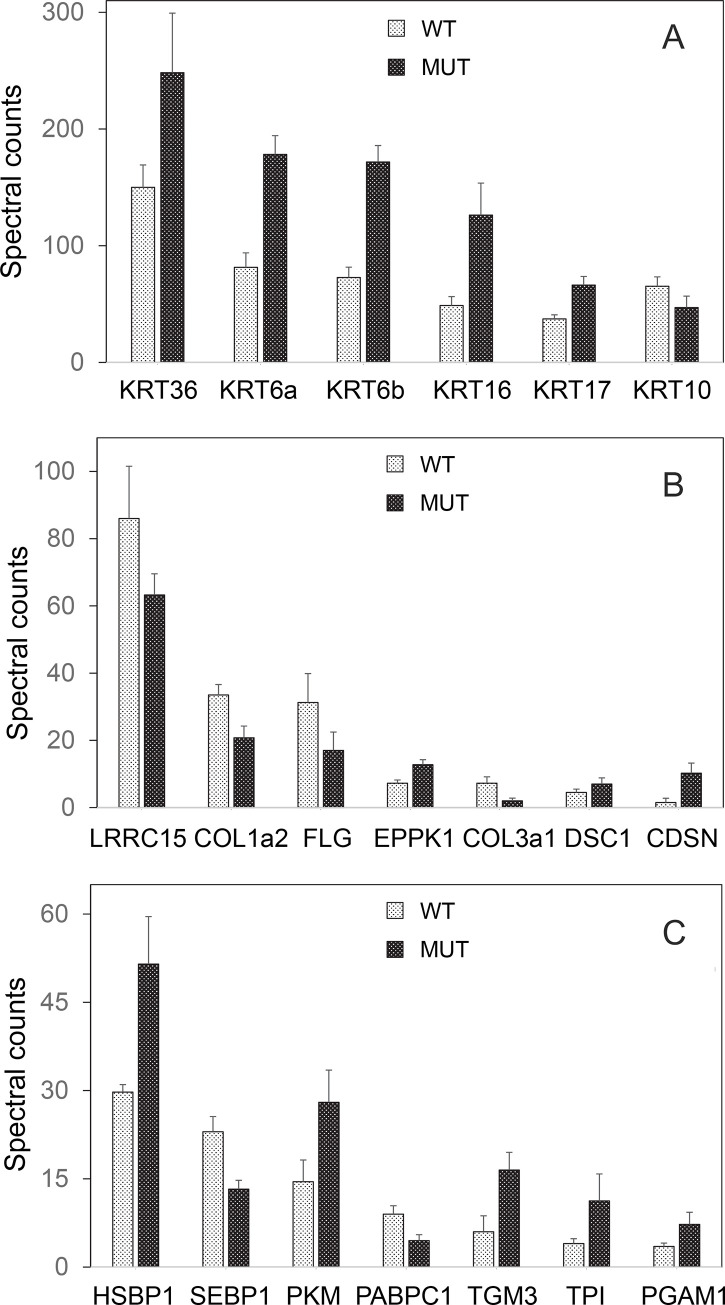
Proteomic analysis. Relative expression levels of 20 representative proteins as judged by spectral counting among those different in mutant (MUT) versus wild type (WT) nail. Illustrated are (A) keratins, (B) structural proteins and (C) binding proteins and enzymes.

To determine to what degree the mutation affected protein expression overall, the profiles of mutant and wildtype nail samples were analyzed. Of the 290 proteins submitted for statistical analysis (S1 Table), 49 were expressed at significantly different levels (28 higher, 21 lower) in the mutant compared to wildtype samples (S2 Table). The degree of difference in expression is illustrated by the 20 proteins with the highest yield of peptides (Fig 8). Among the keratins, 5 (KRT6A, 6B, 16, 17, and 36) were clearly higher in the mutant samples, while one (KRT10) was lower (Fig 8A). Such changes in keratin level are observed as a consequence of a single keratin mutation in human pachyonychia congenita [33]. A variety of structural proteins, including collagens (COL1A2, COL3A1), corneodesmosin (CDSN), desmocollin 1 (DSC1), epiplakin (EPPK1), filaggrin (FLG), and leucine rich repeat protein 15 (LRRC15) showed similar degrees of difference (Fig 8B). The same was also seen (Fig 8C) for heat shock (HSBP1), polyadenylate (PABPC1) and selenium binding proteins (SEBP1) and the enzymes phosphoglycerate mutase 1 (PGAM1), pyruvate kinase (PKM), epidermal transglutaminase (TGM3) and triose phosphate isomerase (TPI).

Notably, KRT90 was not altered significantly in expression, indicating the protein was expressed at near normal levels. However, a clear difference in peptide yields was observed in the samples (Fig 1B). Since the frame-shift mutation produced a different amino acid sequence at the C-terminus, the peptides in that region had no tryptic peptides in common with the wildtype protein and were not detected at all in the database search. The wildtype sequence (Fig 1B) was rich in glycine and serine (each 20 of 63 residues) but, by contrast, the shorter mutant sequence was rich in arginine (12 of 35 residues).

## Discussion

Nail diseases are not frequently reported in mice, probably because they are easily overlooked due to their small size and lack of interest of investigators. A number of spontaneous and genetically engineered mutant mice develop long, overgrown nails early in life. This is a relatively obvious finding. Mice homozygous for mutations in the hairless gene (*Hr*) have long been known to have this phenotype [4]. Genocopies of the hairless phenotype, such as transgenic mice overexpressing ornithine decarboxylase, also have overgrown nails [34]. A common histologic feature of these abnormal nails is the extension of the cornified hyponychium under the nail plate causing separation of the nail plate from the attachment to the nail bed. The region of cornification is associated with extension of the stratum granulosum. These changes were observed in the *Krt90*^*whnl/whnl*^ mutant mice described here. However, unlike these other examples of onychogryphosis, there was a primary anatomical abnormality of the nail bed in which the cells were dystrophic and cornified. Utilization of a monoclonal antibody directed against the horse KRT124 protein yielded an expression pattern in normal mouse nails that matched the location of lesions in these mutant mice. Proteomic analysis of the nail plate indicated that this is not a true null mutation. It is interesting to note that the *Krt90*^*em1(IMPC)J*^ null mutant produced in the International Mouse Phenotyping consortium (IMPC) large scale mutagenesis program website did not report any significant limb, digit, or tail lesions (https://www.mousephenotype.org/, 17 Feb 2021).

No mutations in the human KRT90 gene have been reported as mouse KRT90 is listed as the ortholog to the human KRT90P (keratin 90 pseudogene) (https://rgd.mcw.edu/rgdweb/report/gene/main.html?id=1620571, 9 Dec 2021). No diseases were identified in a Pubmed search nor were there any entries in Online Mendelian Inheritance in Man website (https://www.omim.org/, 9 Dec 2021). Therefore, it is unlikely that this will serve as a model for a specific human disease. However, it does provide a tool to investigate the molecular development of the nail unit and particularly the role of the conserved sequences of unknown function at the C-terminal ends of certain keratins [35]. Moreover, nail unit (hoof) diseases and associated lameness are significant welfare and economic concerns in horses, cows, and other ungulates of agricultural importance [36,37] and KRT90 orthologs (KRT124) have been described in dogs and horses [18,38].

The keratin cytoskeleton is important for keratinocyte stability under mechanical stress. Numerous instances are known where keratin mutations give rise to epidermal fragility syndromes [39,40]. Most attention to keratin structure has focused on the central rod domains that associate to form heterodimers during filament formation, but the C-terminal ends of keratins can have functional importance. The isolated tail of human KRT14 enhances self-organization of KRT5-KRT14 filaments [41], and a translational frame shift near the C-terminus of KRT5, analogous to the presently observed insertion in mouse KRT90, yields a rare form of epidermolysis bullosa simplex [42]. Although not causing fragility, a translational frame shift and premature termination near the C-terminus of KRT1 results in a rare type of ichthyosis [43].

Finding the tissue distribution of KRT90 will be of interest to permit evaluation whether the expression level is sufficient beyond the nail to induce subtle phenotypic alterations that become evident under stress. Since application of proteomic analysis to human nail plate has revealed a profile intermediate between hair shaft and epidermal corneocytes [44], determining expression of KRT90 especially at those two anatomic sites will be of interest. In mouse nail, proteomic analysis has permitted distinguishing proteins subject to remodeling by autophagy during cornification [45]. The degree to which cellular damage due to the present fragility induces degradation of some proteins will also be of interest.

Understanding the specific functions of the C-terminal regions, which can vary greatly in different keratins, remains rudimentary. However, the high content of glycine and sometimes other residues, including serine, has been speculated to serve a structural role such as mediating interactions with other proteins [46]. The large change in sequence of the C-terminus in the *Krt90*^*whnl/whnl*^ mutant mice, where neutral glycine and serine residues are largely replaced by basic arginine residues, could rationalize a perturbation of function by this region. The nail harboring the present KRT90 C-terminal translational frame shift displayed a number of proteins with altered expression levels, which could contribute to the altered phenotype. However, the observed separation of the nail plate from the nail bed and the hyperplastic hyponychium is consistent with undue fragility of the cells. Thus, the most likely explanation for the unusual phenotype is stress-induced cellular damage due to weakness of KRT90-containing intermediate filaments with consequent compensatory hyperplasia of the nail matrix and nail plate.

The equine ortholog of KRT90, KRT124, is the most abundant type II keratin of the equine nail bed [18]. Equine KRT124 expression is restricted to the nail bed and absent from the nail matrix and other surface epithelia, suggesting that this keratin is essential to the function of this highly specialized structure [19]. The adaptation of equids to single-toed unguligrade locomotion requires the strong, but flexible, suspension of the distal phalanx within the hoof capsule. Each single-toed foot of a 500 kg horse must withstand peak ground reaction forces of 2–5,000 N while protecting the underlying limb from trauma [47,48]. The suspension of the distal phalanx within the hoof capsule is mediated by the equine nail bed epithelium, called the hoof lamellae in this species due to its extensive folding into primary and secondary leaves that increase the nail bed surface area to approximately 0.8 m^2^ per hoof [49]. A mouse monoclonal antibody raised against a C-terminal peptide of equine KRT124 labeled the nail bed, matrix, and hyponychium of the normal mouse nail unit, but immunoblots of toe proteins from wildtype mice indicate that this antibody might cross-react with other mouse keratins. KRT42 (formerly KRT17n), the type I keratin partner of KRT90, was localized to the nail matrix and nail bed of mice by *in situ* hybridization [50], suggesting that KRT90 also localizes to these regions, its localization is conserved from rodents to equids, and it is a critical gene for nail unit mechanical function.

The nail unit lesions associated with the *Krt90*^*whnl*^ mutation in mice have many parallels with hoof lesions associated with equine laminitis and demonstrate the importance of nail bed and nail matrix keratins in the form and mechanical function of the nail unit in both species. Without corrective trimming, horses with laminitis develop long curved hooves, although they are dorsally concave rather than convex since nail plate growth is reduced in the dorsal region relative to the sides of the hoof capsule [51]. The nail bed lesions are very similar between the two species and are grossly evident in the laminitic horse as the lamellar wedge, comprised of the abnormal hyperplastic and acanthotic nail bed tissue [15,52] and histologically as epidermal dysplasia, metaplasia, loss of cell-cell and cell-matrix adhesion, hyperplasia, apoptosis, and necrosis [16]. In both the *Krt90*^*whnl*^ mutant mice and horses with laminitis, these lesions can progress to partial or complete sloughing of the nail unit [16,51]. Equine laminitis is also characterized by increased KRT17 expression [53] and abnormal localization of KRT14, which is normally restricted to the basal cell layer of the equine nail bed [54,55]. The similarities in these lesions suggest that, in spite of the difference in initiating cause, several subsequent aspects of equine laminitis pathogenesis may be modeled by the *Krt90*^*whnl*^ mutant mouse. Although the KRT90P pseudogene is not expressed in the human nail unit, the *Krt90*^*whnl*^ mutant mouse can also model human nail bed and nail matrix pathology, particularly the secondary changes associated with conditions that impact the mechanical integrity of the nail unit.

## Supporting information

S1 Table Weighted spectral counts. Trypsin-digested nail samples from the wild type (W) and mutant (M) mice were analyzed by mass spectrometry and tabulated after removal of proteins with a minority of exclusive spectral counts.
(XLSX)

S2 Table Statistical analysis of expression level differences among identified proteins. Weighted counts were compared for nails from the wild type versus mutant mice. FC, fold change (ratio of weighted spectral counts in the mutant to wild type nail). p values were adjusted for multiple comparison testing in column F; values <0.05 are highlighted.
(XLSX)

S1 Raw images Immunoblots of panels A-C were performed 6 times. Representative images were used for the panels.
(PDF)
