# Supplementary material for: Witch Nails (Krt90whnl): A spontaneous mouse mutation affecting nail growth and development
Source: PLoS One. 2022 Nov 14;17(11):e0277284. doi: 10.1371/journal.pone.0277284 (PMC9662738; doi:10.1371/journal.pone.0277284)

S1 Raw Images of Western Blots

All K124 immunoblot images: Immunoblotting performed as described in Materials and Methods using a monoclonal antibody against an N-terminal peptide of equine K124. Relative molecular mass of molecular weight markers indicated to the left, position of MW marker bands marked on autoradiograph film with pen while film aligned with the blot. Autoradiograph film image, no image adjustment. LF: left front limb; LR: left rear limb. Skin: haired skin, dorsal region. Sample details listed for individual blots. Note: Immunoblots 1 and 2 were run just as the COVID-19 pandemic was starting and then the project was paused for one year. These same samples were run again in the final set of four experiments, which also included an equine lamellar tissue positive control sample using newly purchased polyacrylamide gel reagents and buffers.

K124 Immunoblot 1A: Lanes, tissue and total protein load: **1**: Male 1 Skin 5 µg; **2**: Male 1 Skin 10 µg; **3**: Male 1 LR Toes 5 µg; **4**: Male 1 LR Toes 10 µg; **5**: Male 1 LF Toes 5 µg; **6**: Male 1 LF Toes 10 µg; **7**: Male 2 Skin 5 µg; **8**: Male 2 Skin 10 µg; **9**: Male 2 LR Toes 5 µg; **10**: Male 2 LR Toes 10 µg; **11**: Male 2 LF Toes 5 µg; **12**: Male 2 LF Toes 10 µg. 1 min exposure.

K124 Immunoblot 2A: Lanes, tissue and total protein load: **1**: Male 3 Skin 10 µg; **2**: Male 3 LR Toes 5 µg; **3**: Male 3 LF Toes 5 µg; **4**: Male 4 Skin 10 µg; **5**: Male 4 LR Toes 5 µg; **6**: Male 4 LF Toes 5 µg; **7**: Male 5 Skin 10 µg; **8**: Male 5 LR Toes 5 µg; **9**: Male 5 LF Toes 5 µg; **10**: Male 6 Skin 10 µg; **11**: Male 6 LR Toes 5 µg; **12**: Male 6 LF Toes 5 µg. 30 sec exposure.

K124 Immunoblot 3A: Lanes, tissue and total protein load: **1**: Male 3 Skin 10 µg; **2**: Male 3 LR Toes 5 µg; **3**: Male 3 LF Toes 5 µg; **4**: Male 4 Skin 10 µg; **5**: Male 4 LR Toes 5 µg; **6**: Male 4 LF Toes 5 µg; **7**: Male 5 Skin 10 µg; **8**: Male 5 LR Toes 5 µg; **9**: Male 5 LF Toes 5 µg; **10**: Blank; **11**: Equine lamellar tissue 2 µg. 5 min exposure

K124 Immunoblot 4A: Lanes, tissue and total protein load: **1**: Female 1 Skin 10 µg; **2**: Female 1 LR Toes 10 µg; **3**: Female 1 LF Toes 10 µg; **4**: Female 2 Skin 10 µg; **5**: Female 2 LR Toes 10 µg; **6**: Female 2 LF Toes 10 µg; **7**: Female 3 Skin 10 µg; **8**: Female 3 LR Toes 10 µg; **9**: Female 3 LF Toes 10 µg; **10**: Blank; **11**: Equine lamellar tissue 2 µg. 1 min exposure.

K124 Immunoblot 5A: Lanes, tissue and total protein load: **1**: Female 4 Skin 10 µg; **2**: Female 4 LR Toes 10 µg; **3**: Female 4 LF Toes 10 µg; **4**: Female 5 Skin 10 µg; **5**: Female 5 LR Toes 10 µg; **6**: Female 5 LF Toes 10 µg; **7**: Female 6 Skin 10 µg; **8**: Female 6 LR Toes 10 µg; **9**: Female 6 LF Toes 10 µg; **10**: Blank; **11**: Equine lamellar tissue 1 µg. 5 min exposure

K124 Immunoblot 6A: Lanes, tissue and total protein load: **1**: Male 1 Skin 10 µg; **2**: Male 1 LR Toes 10 µg; **3**: Male 1 LF Toes 10 µg; **4**: Male 2 Skin 10 µg; **5**: Male 2 LR Toes 10 µg; **6**: Male 2 LF Toes 10 µg; **7**: Male 6 Skin 10 µg; **8**: Male 6 LR Toes 10 µg; **9**: Male 6 LF Toes 10 µg; **10**: Blank; **11**: Equine lamellar tissue 1 µg. 30 sec exposure. Note: Part of this immunoblot is shown in Fig 7, panel A.

K124 Immunoblot 6B: Same blot as 6A, secondary antibody alone control prior to primary antibody blotting. 1 min exposure. Blot was then stripped and reprobed with K124 antibody (6A). Note: Part of this immunoblot is shown in Fig 7, panel D.

All K14 Immunoblot images: K124 Western Blot 1A stripped and blocked overnight with anti-K14 antibody and anti-β-actin antibody, as described in Materials and Methods. The 50 kDa band is K14 and the 42 kDa band is β-actin.

K14 Immunoblot 1B: Lanes, tissue and total protein load: **1**: Male 1 Skin 5 µg; **2**: Male 1 Skin 10 µg; **3**: Male 1 LR Toes 5 µg; **4**: Male 1 LR Toes 10 µg; **5**: Male 1 LF Toes 5 µg; **6**: Male 1 LF Toes 10 µg; **7**: Male 2 Skin 5 µg; **8**: Male 2 Skin 10 µg; **9**: Male 2 LR Toes 5 µg; **10**: Male 2 LR Toes 10 µg; **11**: Male 2 LF Toes 5 µg; **12**: Male 2 LF Toes 10 µg. 30 sec exposure.

K14 Immunoblot 2B: Lanes, tissue and total protein load: **1**: Male 3 Skin 10 µg; **2**: Male 3 LR Toes 5 µg; **3**: Male 3 LF Toes 5 µg; **4**: Male 4 Skin 10 µg; **5**: Male 4 LR Toes 5 µg; **6**: Male 4 LF Toes 5 µg; **7**: Male 5 Skin 10 µg; **8**: Male 5 LR Toes 5 µg; **9**: Male 5 LF Toes 5 µg; **10**: Male 6 Skin 10 µg; **11**: Male 6 LR Toes 5 µg; **12**: Male 6 LF Toes 5 µg. 1 min exposure.

K14 Immunoblot 3B: Lanes, tissue and total protein load: **1**: Male 3 Skin 10 µg; **2**: Male 3 LR Toes 5 µg; **3**: Male 3 LF Toes 5 µg; **4**: Male 4 Skin 10 µg; **5**: Male 4 LR Toes 5 µg; **6**: Male 4 LF Toes 5 µg; **7**: Male 5 Skin 10 µg; **8**: Male 5 LR Toes 5 µg; **9**: Male 5 LF Toes 5 µg; **10**: Blank; **11**: Equine lamellar tissue 2 µg. 1 min exposure

K14 Immunoblot 4B: Lanes, tissue and total protein load: **1**: Female 1 Skin 10 µg; **2**: Female 1 LR Toes 10 µg; **3**: Female 1 LF Toes 10 µg; **4**: Female 2 Skin 10 µg; **5**: Female 2 LR Toes 10 µg; **6**: Female 2 LF Toes 10 µg; **7**: Female 3 Skin 10 µg; **8**: Female 3 LR Toes 10 µg; **9**: Female 3 LF Toes 10 µg; **10**: Blank; **11**: Equine lamellar tissue 2 µg. 30 sec exposure.

K14 Immunoblot 5B: Lanes, tissue and total protein load: **1**: Female 4 Skin 10 µg; **2**: Female 4 LR Toes 10 µg; **3**: Female 4 LF Toes 10 µg; **4**: Female 5 Skin 10 µg; **5**: Female 5 LR Toes 10 µg; **6**: Female 5 LF Toes 10 µg; **7**: Female 6 Skin 10 µg; **8**: Female 6 LR Toes 10 µg; **9**: Female 6 LF Toes 10 µg; **10**: Blank; **11**: Equine lamellar tissue 1 µg. 30 sec exposure

K14 Immunoblot 6C: Lanes, tissue and total protein load: **1**: Male 1 Skin 10 µg; **2**: Male 1 LR Toes 10 µg; **3**: Male 1 LF Toes 10 µg; **4**: Male 2 Skin 10 µg; **5**: Male 2 LR Toes 10 µg; **6**: Male 2 LF Toes 10 µg; **7**: Male 6 Skin 10 µg; **8**: Male 6 LR Toes 10 µg; **9**: Male 6 LF Toes 10 µg; **10**: Blank; **11**: Equine lamellar tissue 1 µg. 30 sec exposure. Note: Part of this immunoblot is shown in Fig 7, panel B.

Amido Black Protein Stain images: Shown for Blots 1-6, protein stain performed as described in Materials and Methods. Blot 6 is also shown in Fig 7, panel C.

K124 Blot 1A

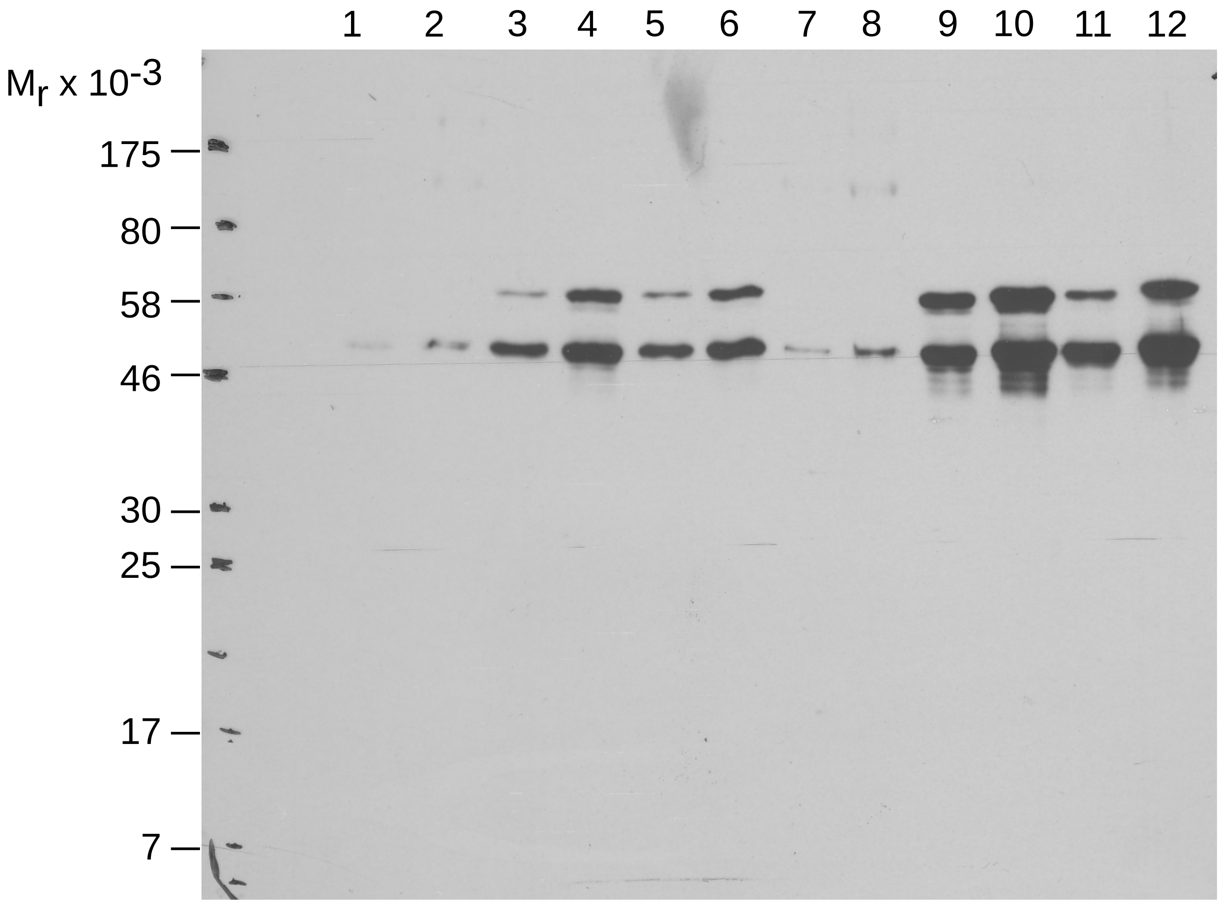

K124 Blot 2A

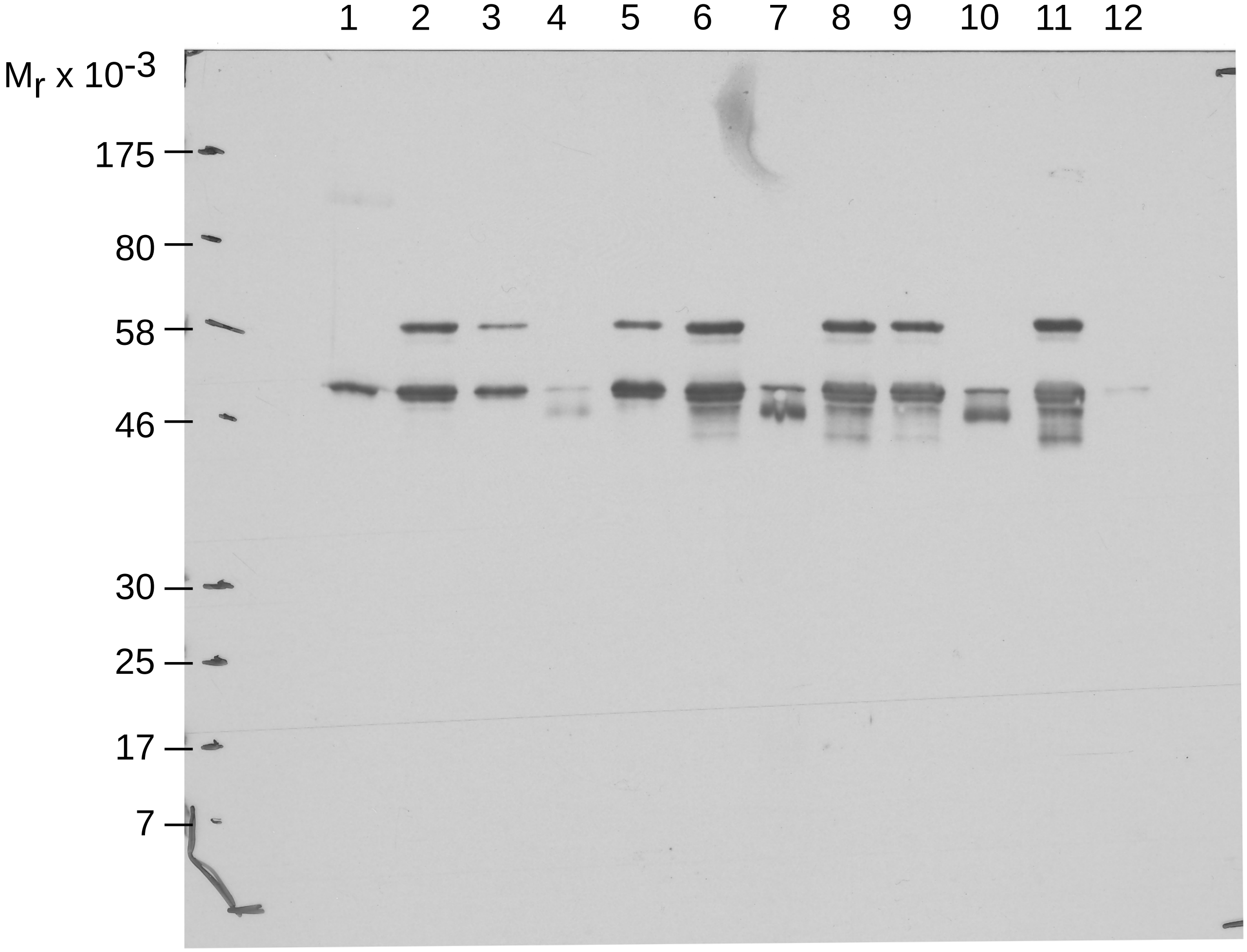

K124 Blot 3A

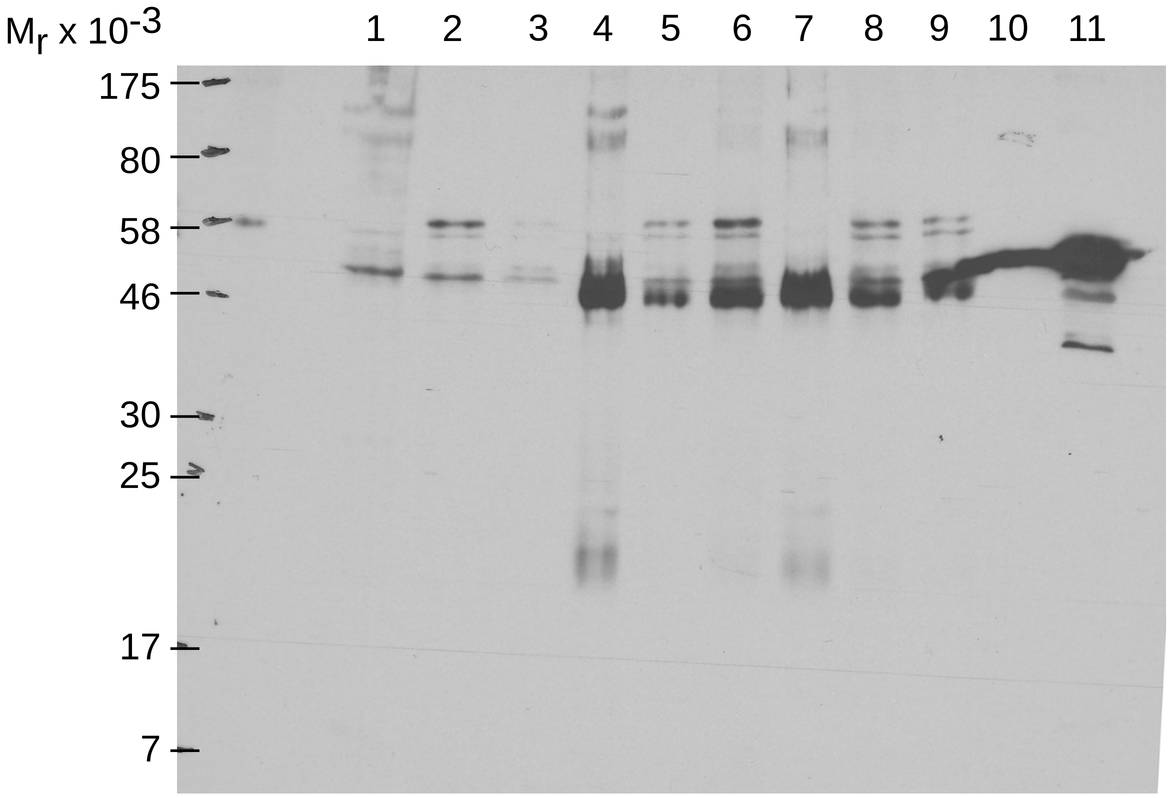

K124 Blot 4A

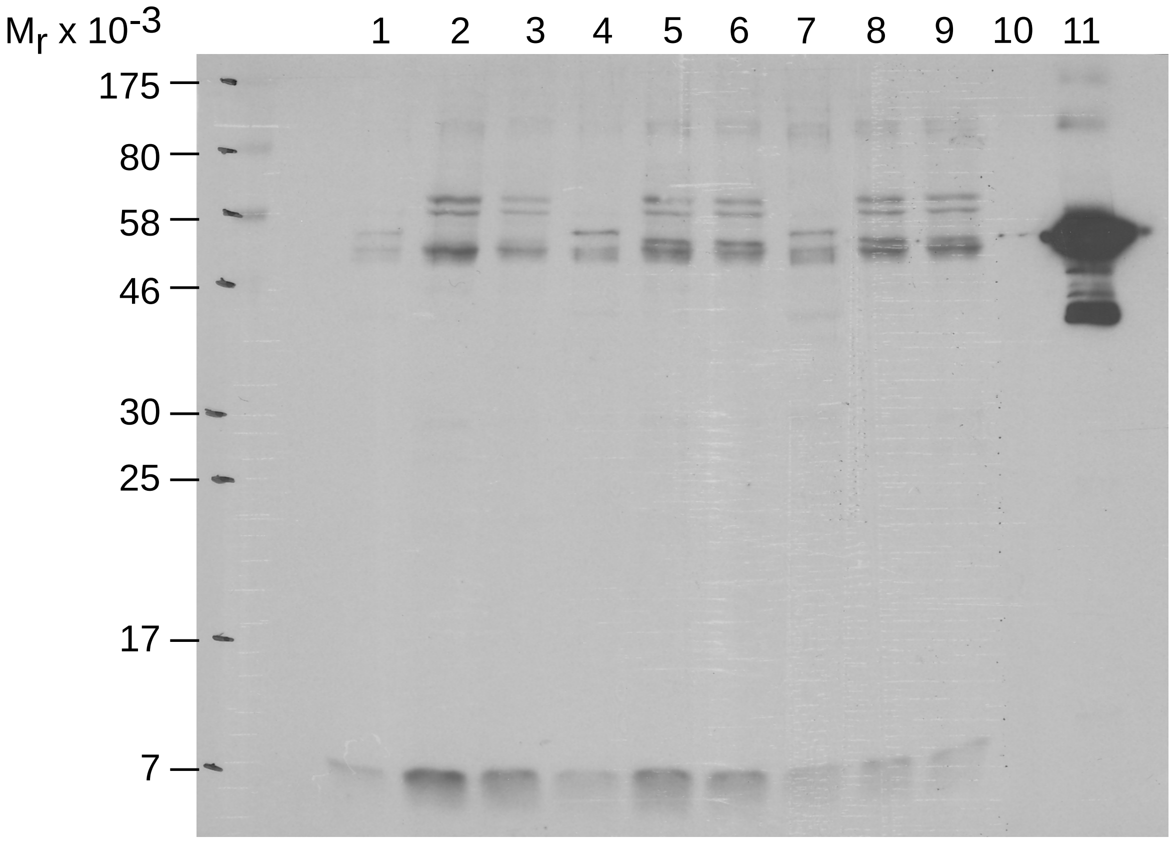

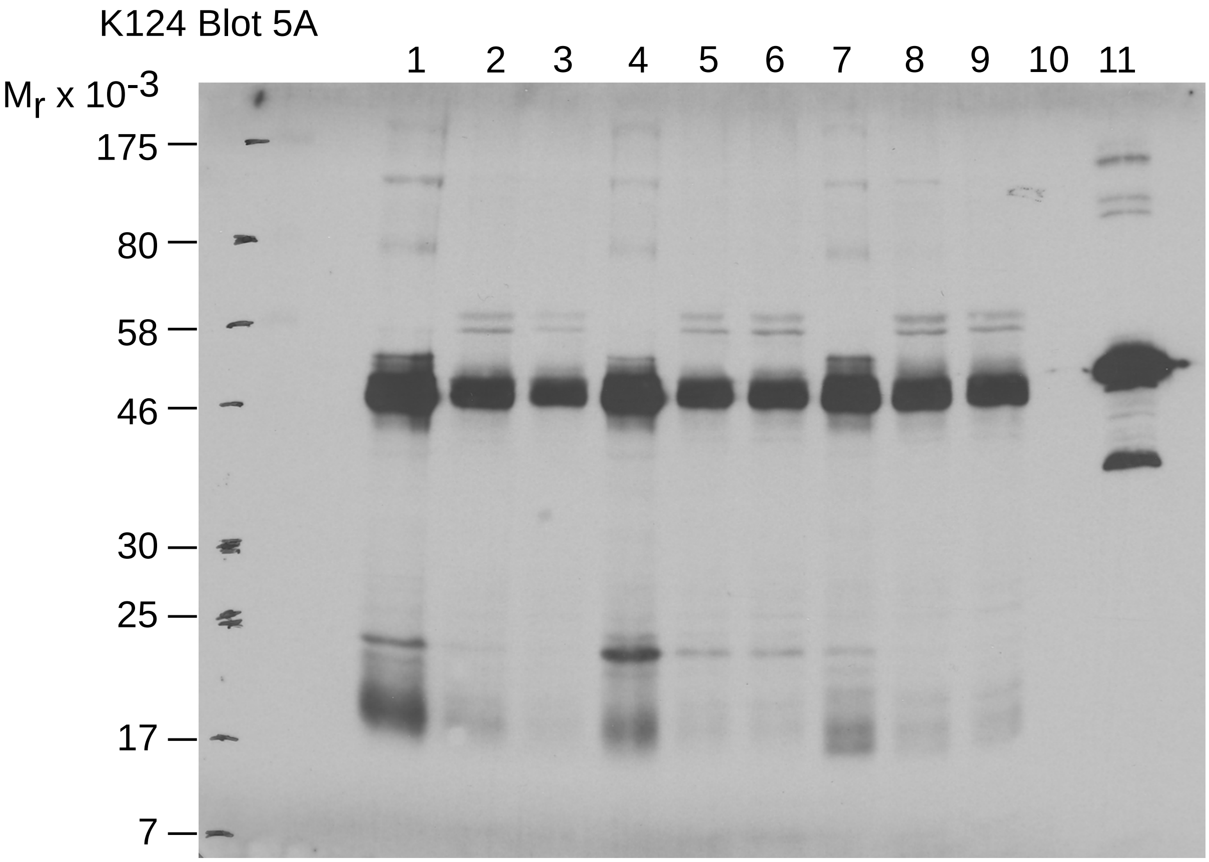
K124 Blot 5A
M_r x 10^{-3}
1 2 3 4 5 6 7 8 9 10 11
175
80
58
46
30
25
17
7

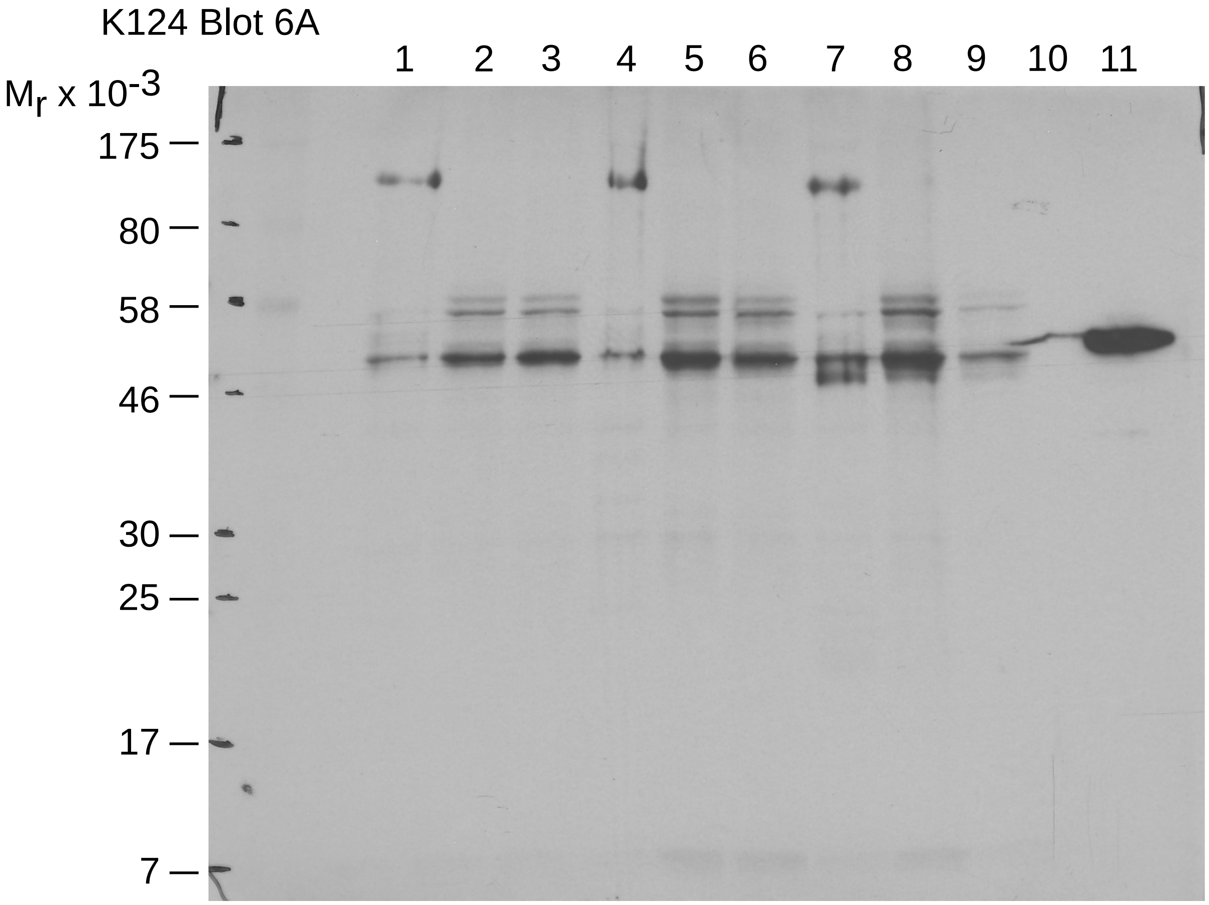
K124 Blot 6A
1 2 3 4 5 6 7 8 9 10 11
$M_r \times 10^{-3}$
175
80
58
46
30
25
17
7

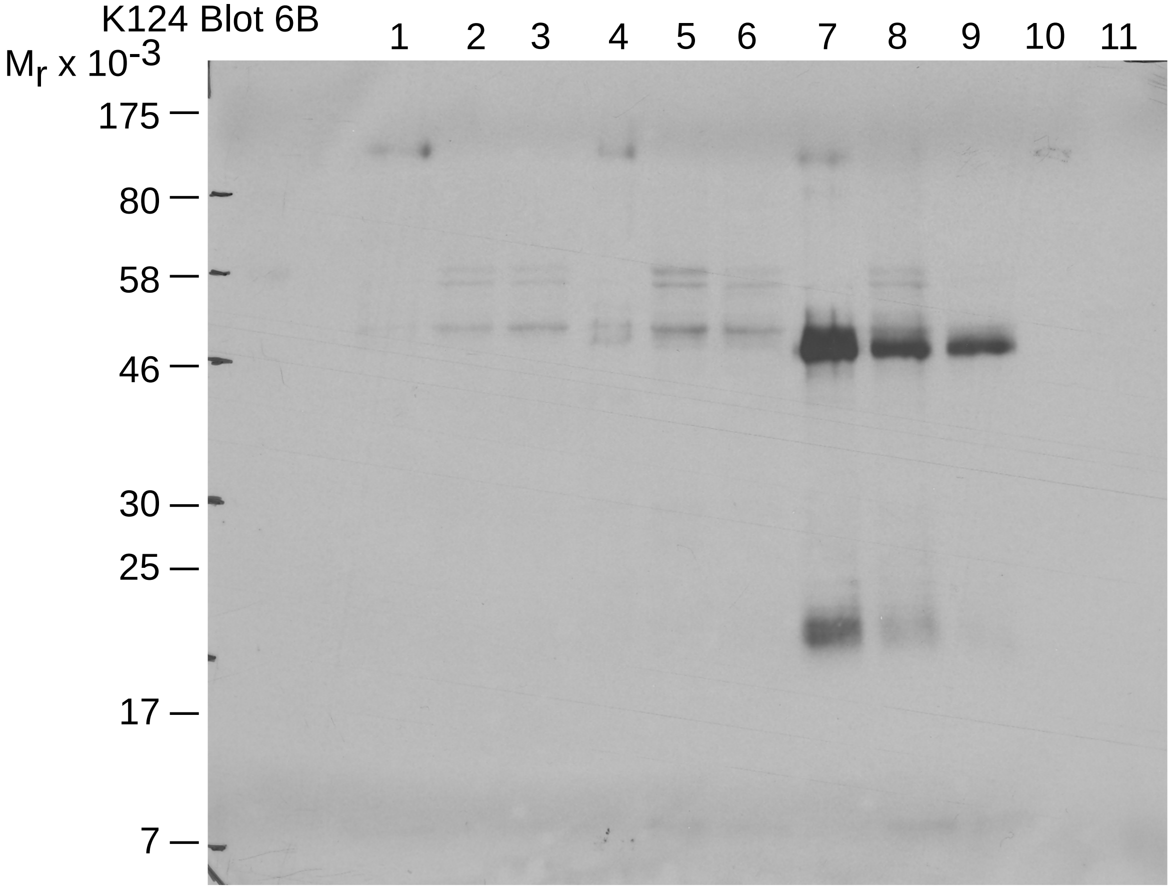
K124 Blot 6B
1 2 3 4 5 6 7 8 9 10 11
$M_r \times 10^{-3}$
175
80
58
46
30
25
17
7

K14 Blot 1B

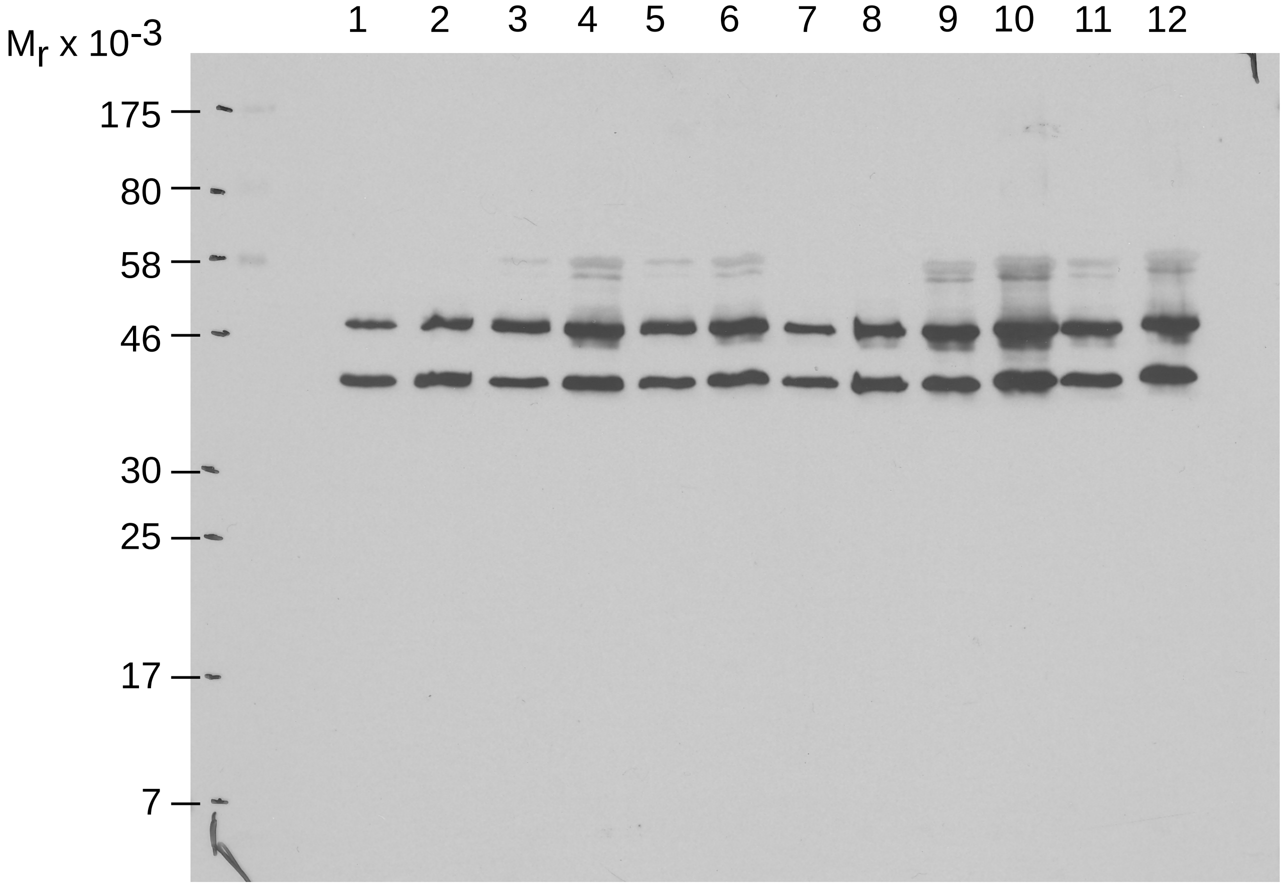

K14 Blot 2B

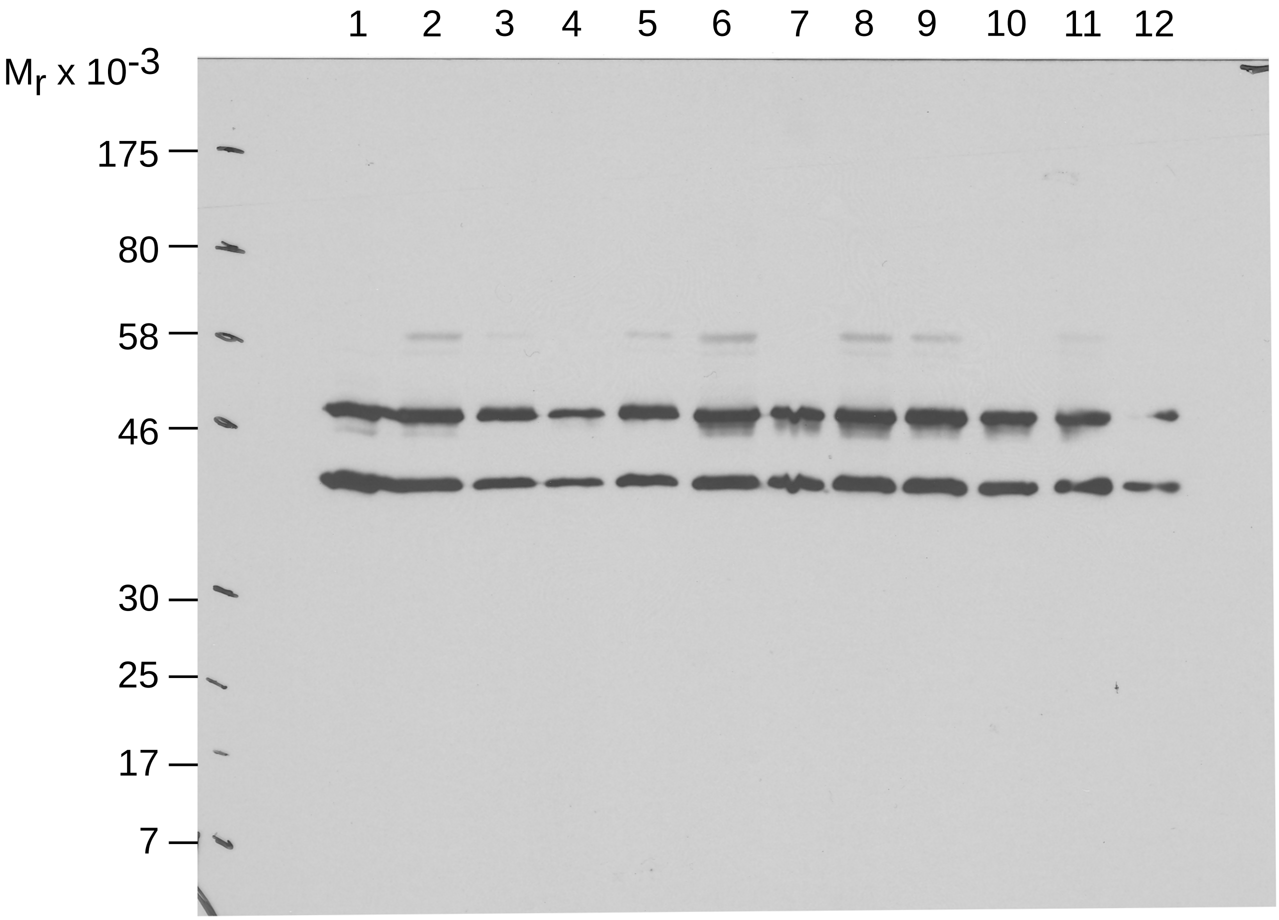

K14 Blot 3B

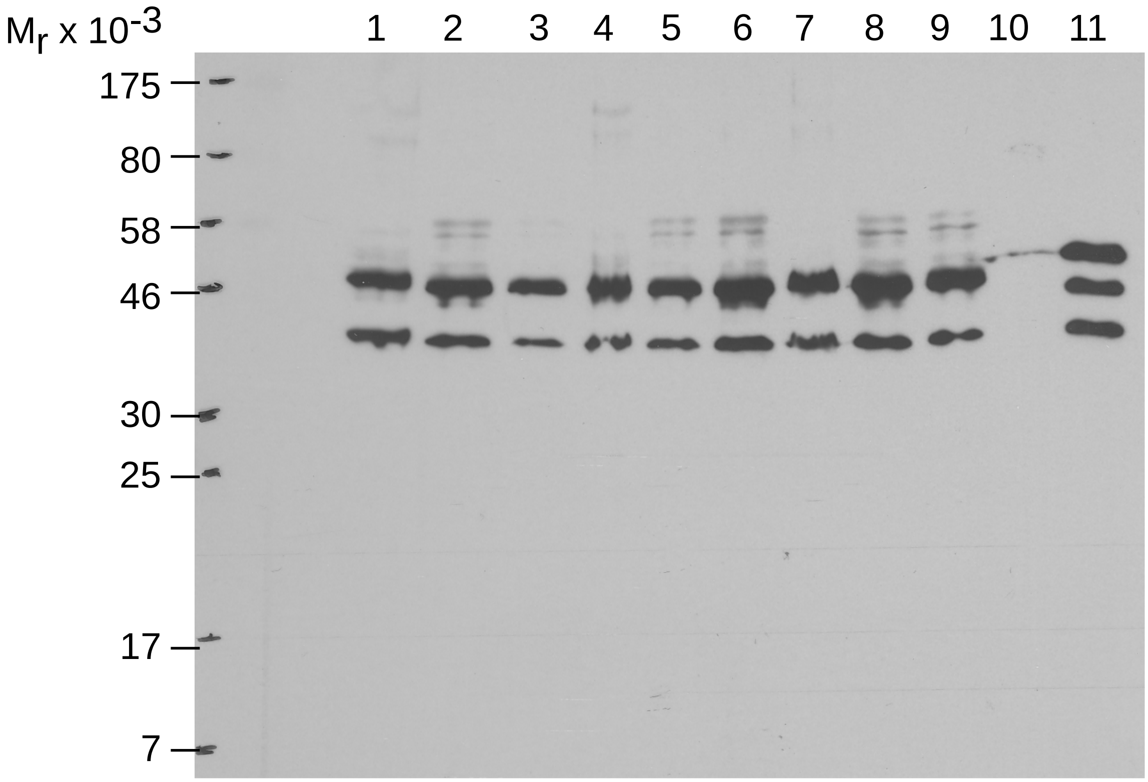

K14 Blot 4B

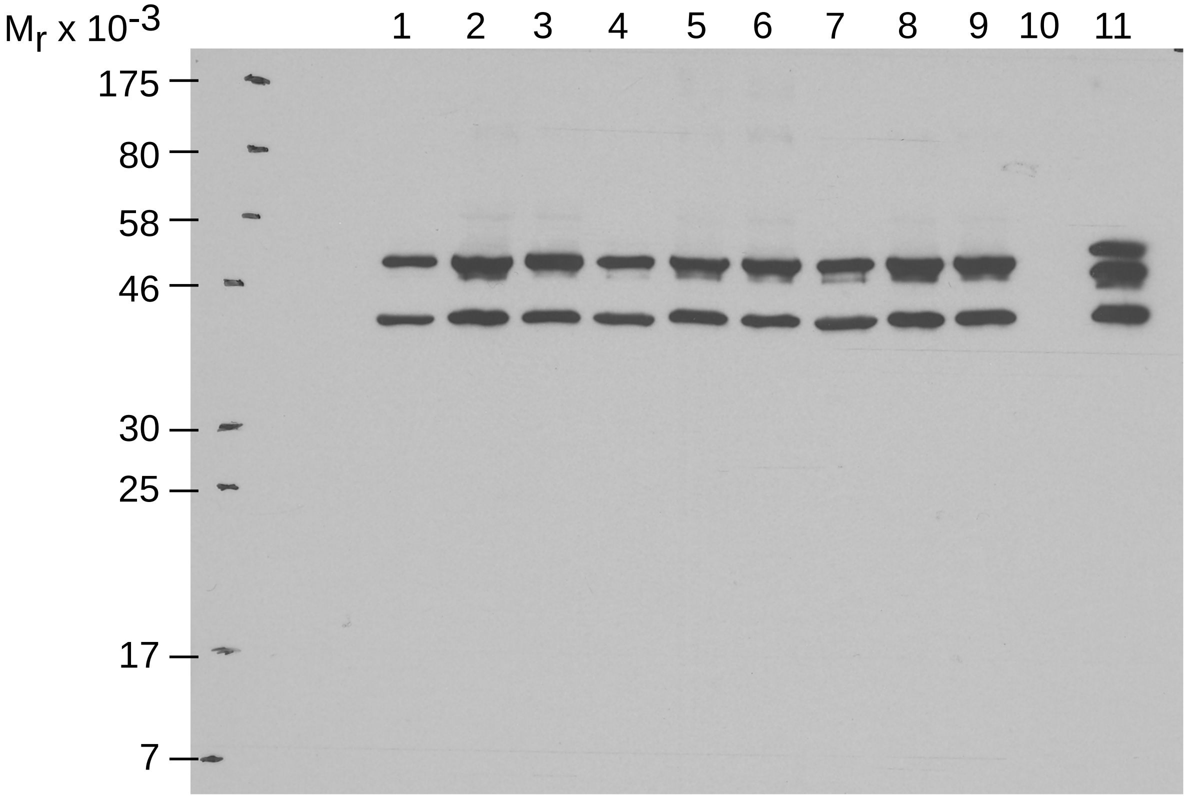

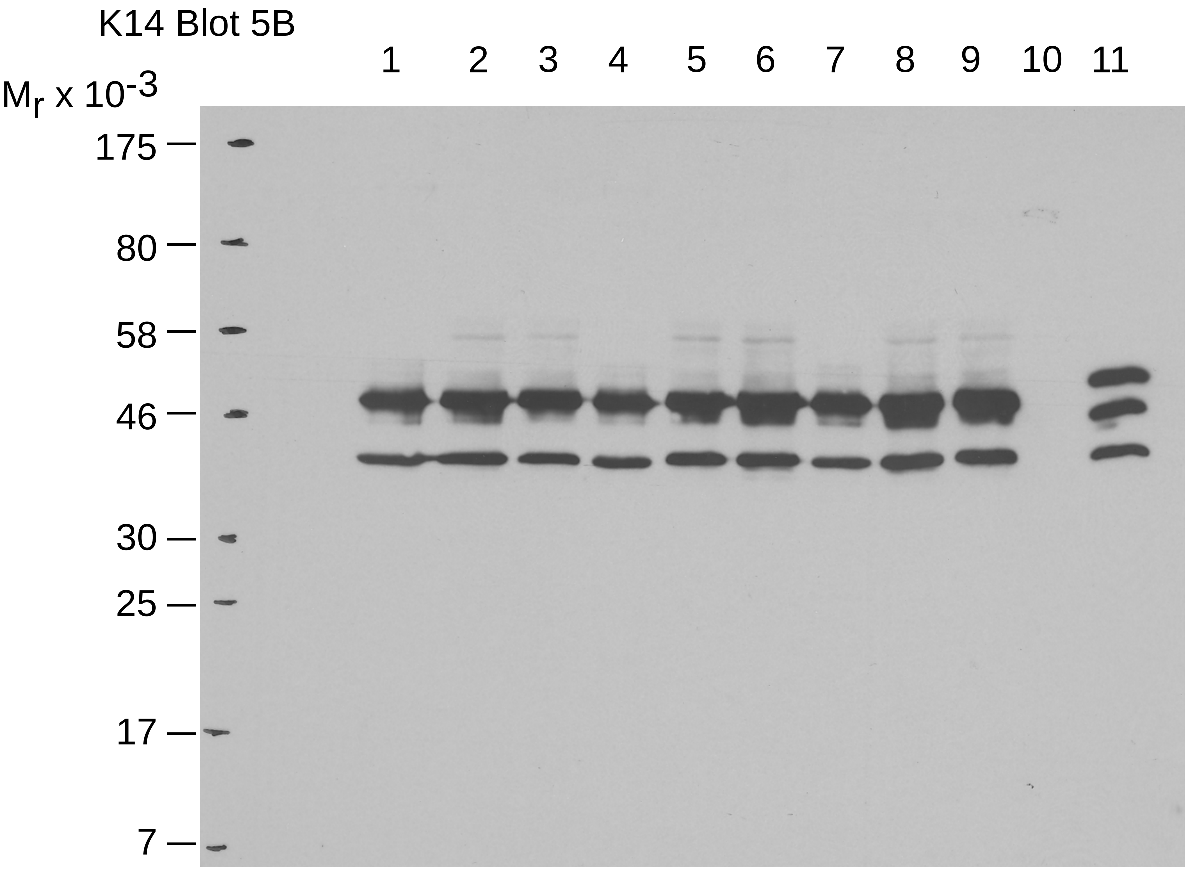
K14 Blot 5B
M_r x 10^{-3}
1 2 3 4 5 6 7 8 9 10 11
175
80
58
46
30
25
17
7

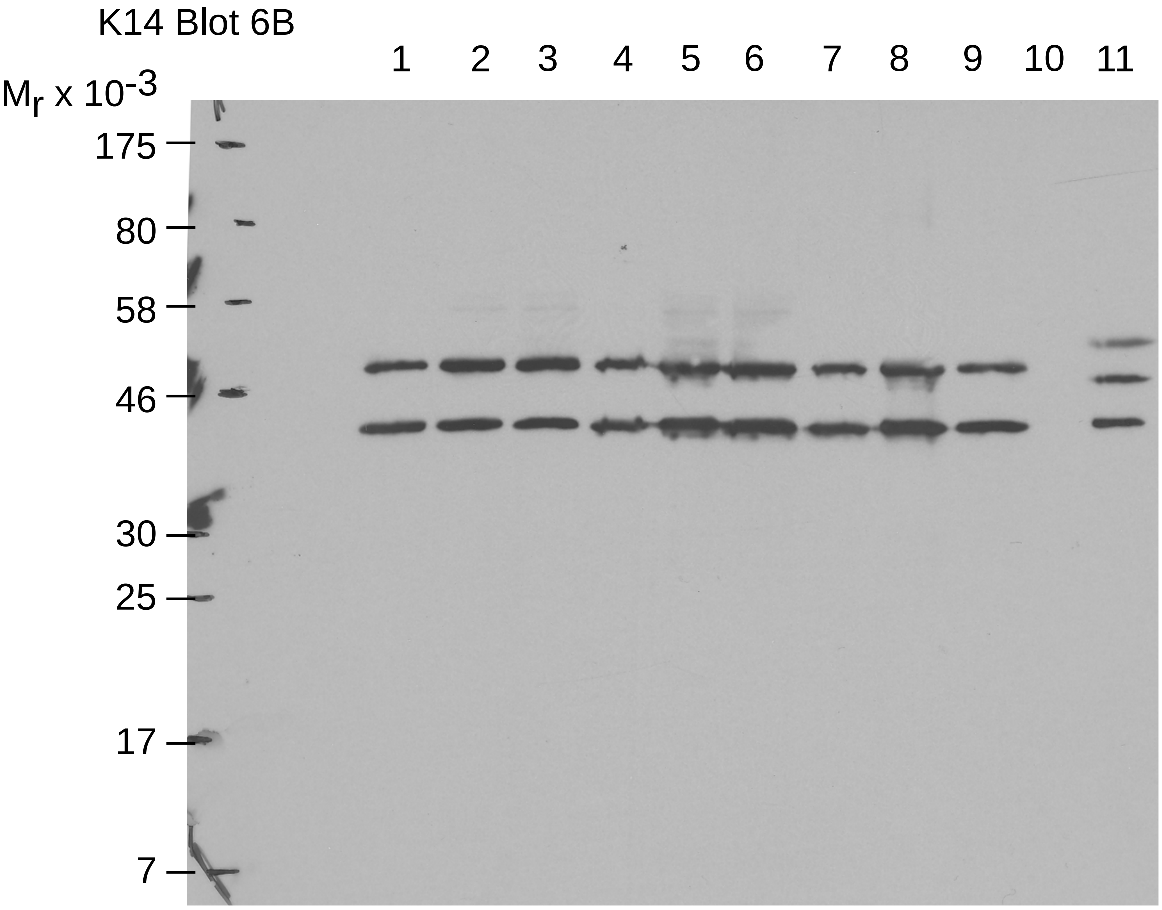

K14 Blot 6B
1
2
3
4
5
6
7
8
9
10
11
$M_r \times 10^{-3}$
175
80
58
46
30
25
17
7

Amido Black Protein Stain Blot 1

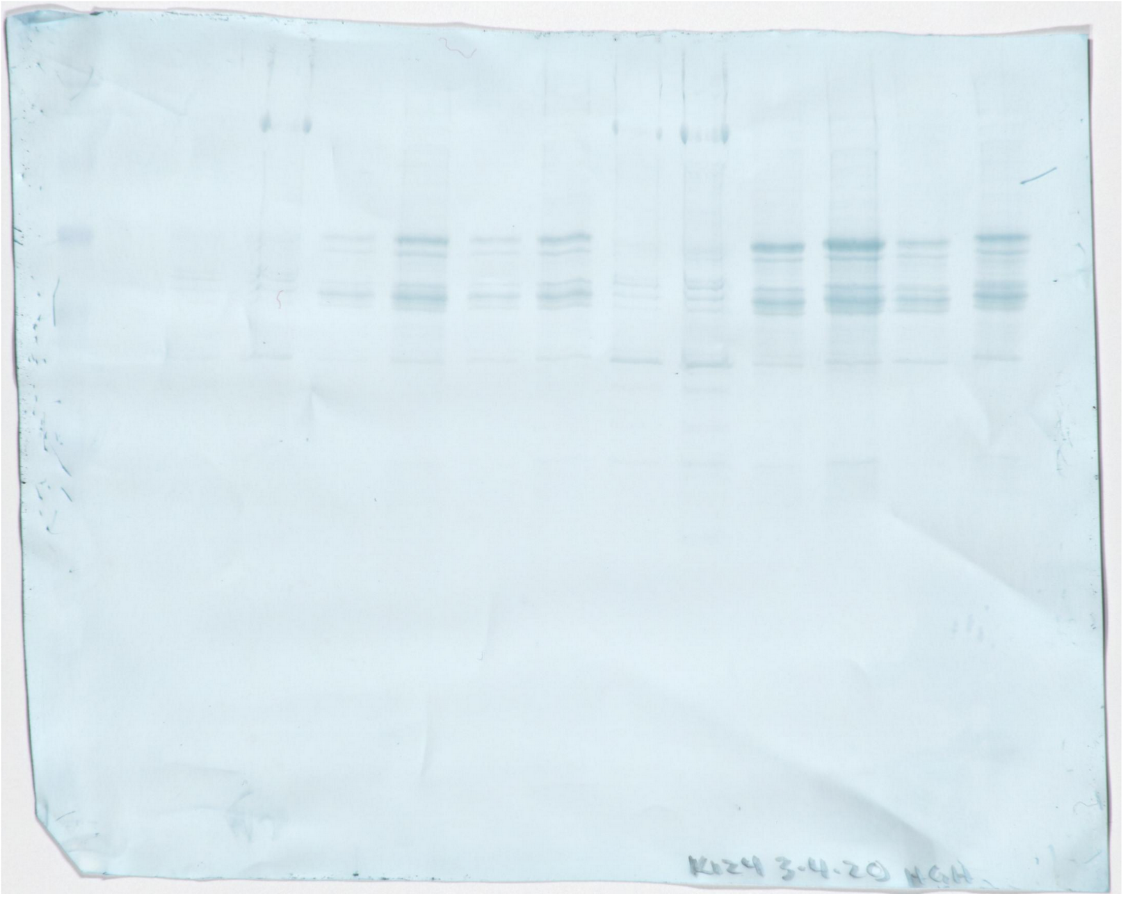

Amido Black Protein Stain Blot 2

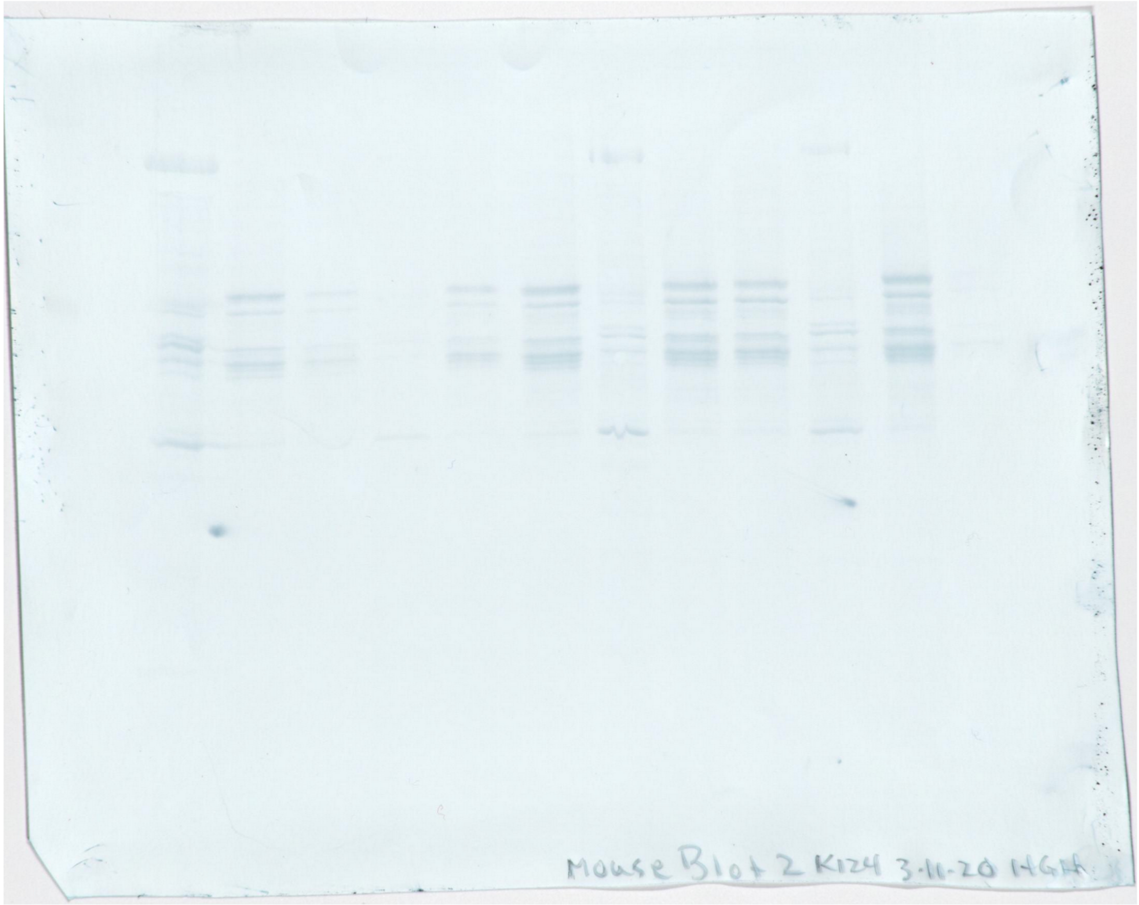

Amido Black Protein Stain Blot 3

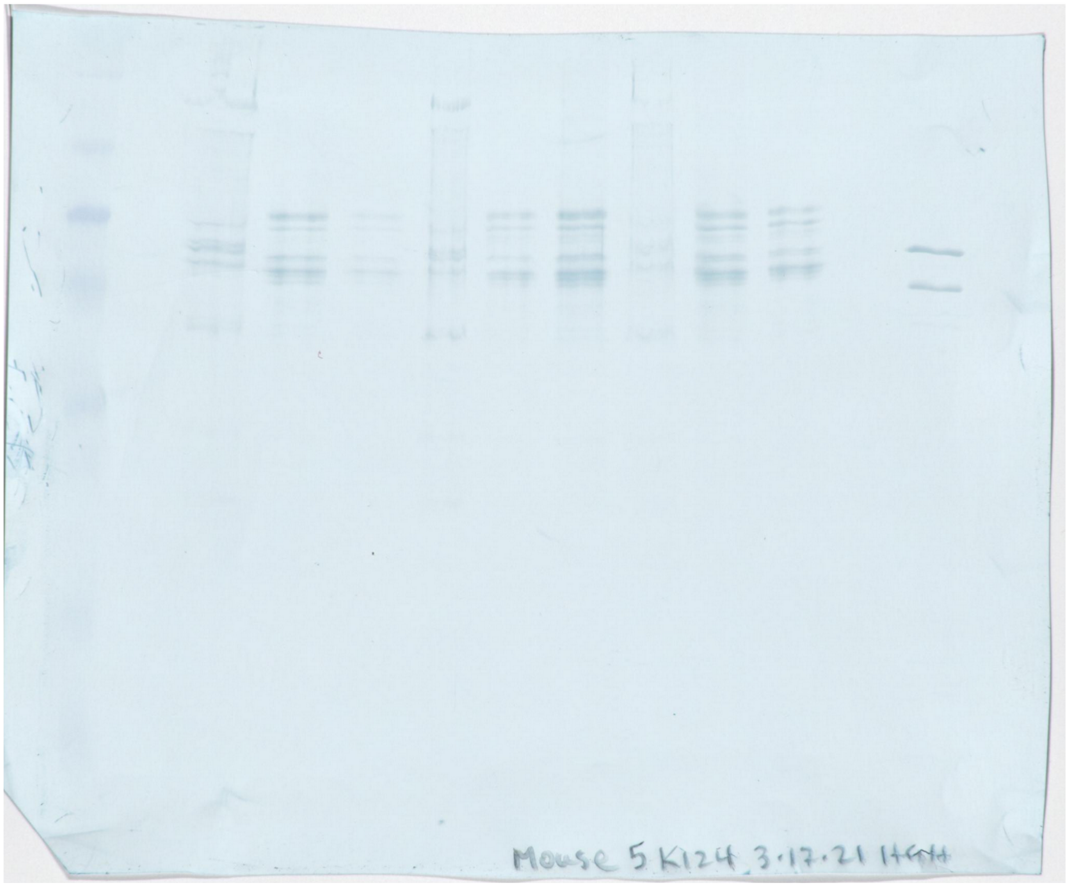

Amido Black Protein Stain Blot 4

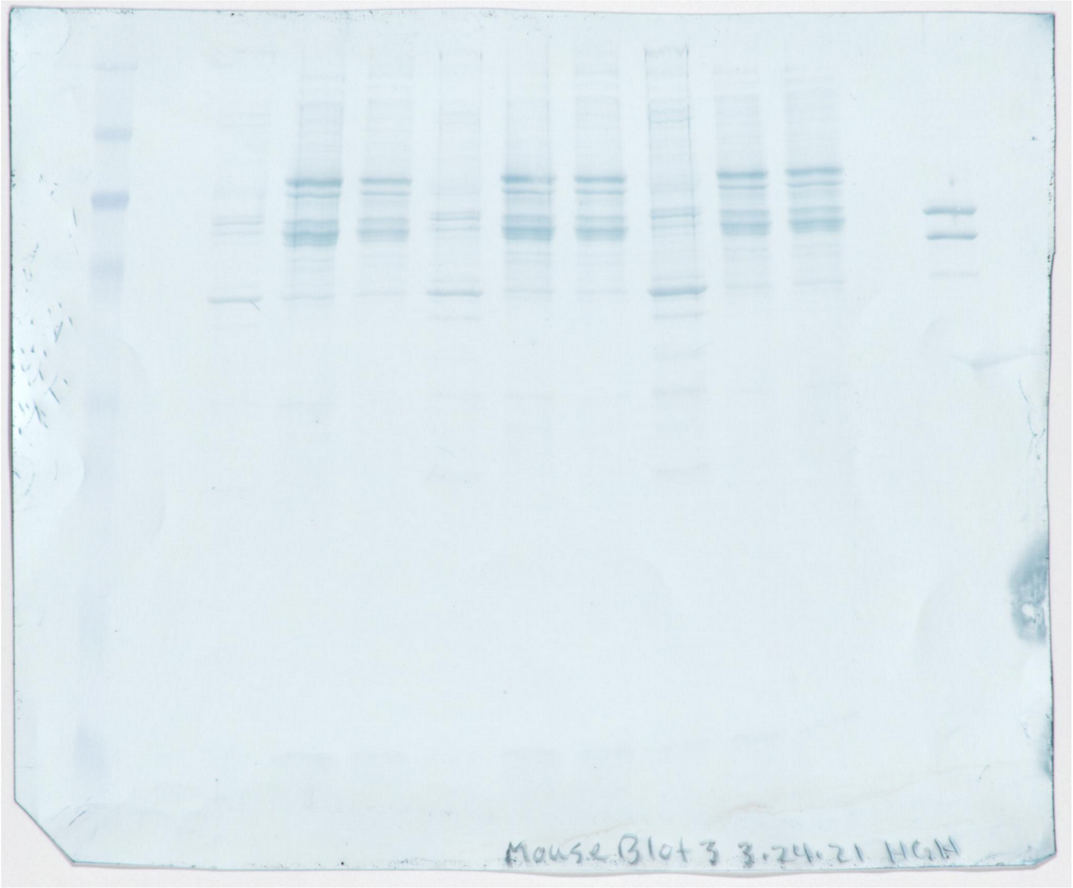

Amido Black Protein Stain Blot 5

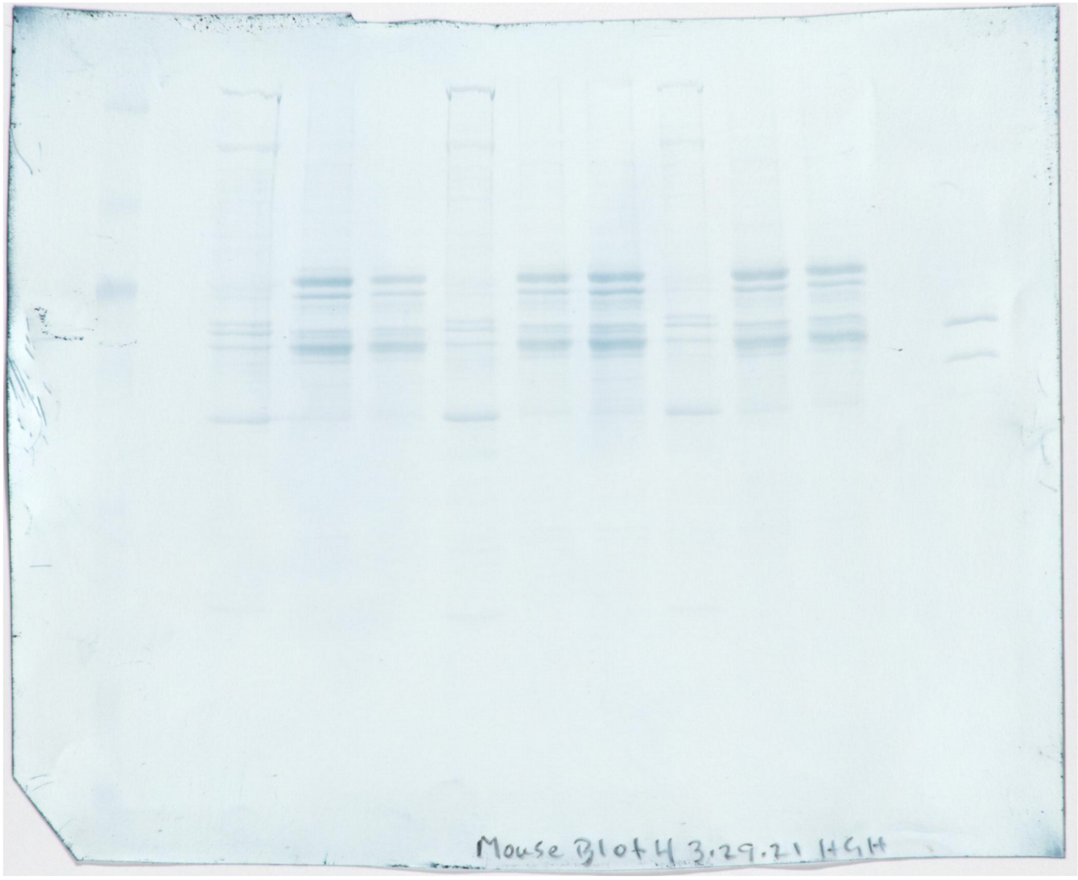

Amido Black Protein Stain Blot 6

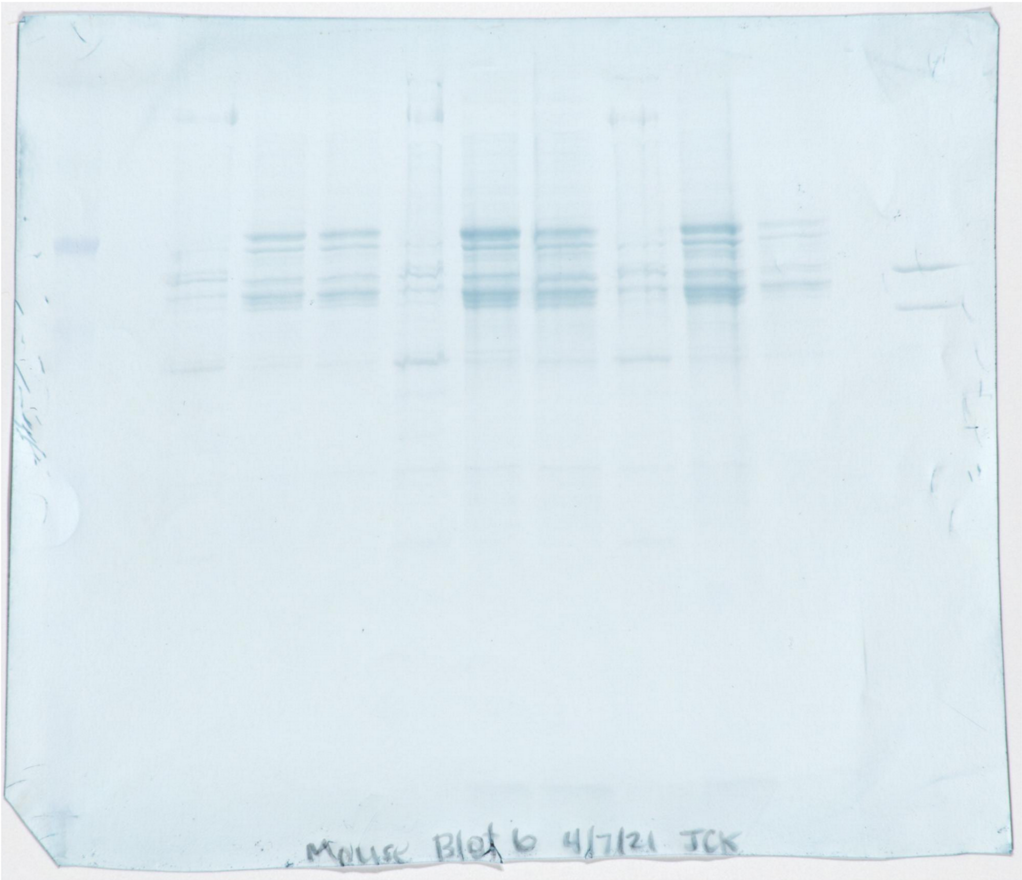

Supplement: S1 Raw images — Representative images were used for the panels. (PDF) [file pone.0277284.s003.pdf]
